# Malaria shaped human spatial organization for the past 74 thousand years

**DOI:** 10.1126/sciadv.aea2316

**Published:** 2026-04-22

**Authors:** Margherita Colucci, Michela Leonardi, James Blinkhorn, Seth R. Irish, Cecilia Padilla-Iglesias, Stefanie Kaboth-Bar, William D. Gosling, Robert W. Snow, Andrea Manica, Eleanor M. L. Scerri

**Affiliations:** ^1^ Human Palaeosystems Group, Max-Planck Institute of Geoanthropology, Jena 07743, Germany.; ^2^ Evolutionary Ecology Group, Department of Zoology, University of Cambridge, Cambridge CB2 3EJ, UK.; ^3^ Natural History Museum, London SW7 5BD, UK.; ^4^ Department of Archaeology, Classics and Egyptology, University of Liverpool, Liverpool L69 7WZ, UK.; ^5^ Malaria and Neglected Tropical Disease Department, World Health Organization, Geneva 1211, Switzerland.; ^6^ Swiss Tropical and Public Health Institute (Swiss TPH), Allschwil 4123, Switzerland.; ^7^ Emmanuel College, University of Cambridge, Cambridge CB2 3AP, UK.; ^8^ Institute of Geological Sciences, Freie Universität Berlin, Berlin 12249, Germany.; ^9^ Institute of Biodiversity and Ecosystem Dynamics, University of Amsterdam, Amsterdam 1090, Netherlands.; ^10^ Kenya Medical Research Institute (KEMRI)–Wellcome Trust Research Programme, Nairobi 80108-230, Kenya.; ^11^ Centre for Tropical Medicine and Global Health, Nuffield Department of Clinical Medicine, University of Oxford, Oxford OX3 7LG, UK.; ^12^ Department of Classics and Archaeology, University of Malta, Msida MSD 2080, Malta.; ^13^ Department of Prehistoric Archaeology, University of Cologne, Cologne 50931, Germany.

## Abstract

While climate is often seen as the main driver of early humans’ spatial organization in Africa, genetic and archaeological studies also suggest diseases as key selective forces in the Pleistocene. We explored whether *Plasmodium falciparum*–induced malaria drove habitat choice in human societies 74,000 to 5000 years ago. Combining species distribution models of mosquito complexes, palaeoclimatic and epidemiological data, we estimated an index of malaria transmission risk in sub-Saharan Africa through time. We then correlated it with an independent reconstruction of the human niche, demonstrating that humans avoided or were unsuccessful in potential malaria hotspots. Our results highlight the importance of considering disease distributions when modeling past human demography, demonstrating that factors beyond climate underlay population structure, patterns of habitat choice, and dispersal.

## Teaser

Malaria has shaped human habitat choice, exchange, and dispersal since the late Pleistocene in sub-Saharan Africa.

## INTRODUCTION

Converging evidence demonstrates that our species, *Homo sapiens* did not have one single birthplace in Africa (*1*–*3*). Instead, the earliest members of our species were divided into small populations that spread across much of the African continent, presenting a markedly different scenario to long-held perceptions of a single center of endemism (supplementary text 1) (*1*–*3*). This paradigm shift implicates more than one region and environment in Africa at the root of our species (*3*) and a recognition that humans occupy a “generalist specialist” niche (*4*). Evidence demonstrating that humans, as a species, occupied a wide range of environments from an early stage while being highly locally adapted at subpopulations level is increasingly apparent [e.g., (*5*)]. As a result, considerable focus has been given to identify the climate mechanisms of population spread/isolation and ecological adaptation in this context [e.g.,(*6*, *7*)]. However, it was not just humans who adapted to different regions and environments, their pathogens did too. In the current race to elucidate processes of human adaptation and spread, there has been little focus on the link between climate and disease and the subsequent impact on human selective processes (*8*, *9*). What was the burden of disease in the earliest periods of our species’ prehistory? How did diseases affect human behavior and demography? How did these factors interact and affected the mixings and dispersals that cumulatively shaped the course of human evolution and, ultimately, the history of all contemporary populations?

Here, we aim to address these questions by studying the impact of diseases in the human past. In particular, we explore how *Plasmodium falciparum*–induced malaria shaped the history of our species in sub-Saharan Africa between 74 thousand and 5 thousand years ago (ka). This period spans substantial demographic expansions within and out of Africa by hunter-gatherer populations (*1*, *7*, *10*–*12*), up until the time shortly before the expansion of farming lifeways (*13*–*16*).

Malaria is a major world disease that today presents a global health problem, with 263 million cases annually (*17*). Critically, genetic studies also indicate that malaria was a major problem both in recent prehistory (*18*) and also in the Pleistocene, with mutations relating to sickle cell anemia emerging in response to *P. falciparum*–induced malaria between 25 and 22 ka in Africa [(*9*); see supplementary text]. Archaeological studies have also identified earlier, indirect evidence for the measures that humans took to avoid exposure to the vectors of disease, for example, by topping plant bedding with aromatic leaves containing insecticidal and larvicidal chemicals (*19*). Other data may suggest an avoidance of certain localities. For example, the absence of sites near major North African rivers during peak periods of the Last Interglacial (~125 to 71 ka) may potentially indicate the avoidance of swampy regions where mosquitos (and other parasites and vectors) thrived (*20*).

Given the indications from genetic and archaeological data that malaria may have shaped early human settlement patterns in sub-Saharan Africa, we reconstructed its distribution over time. Reconstructing past disease incidence and its effects on humans is an endeavor that has typically been challenging due to the limited direct evidence from such remote times (see supplementary text). We overcame the lack of direct evidence by first quantifying the niche of malaria’s main vectors, which, in turn, allowed us to reconstruct their potential spatial distribution for a given point in time based on the climatic conditions. This is done by building species distribution models (SDMs) (*21*) based on present-day occurrences and location-specific environmental and climatic variables and then projecting their ranges back in the past. By combining the obtained vectors’ habitat suitability with epidemiological information (see Materials and Methods), we then calculated an index of potential risk of *P. falciparum*–induced malaria transmission, defined as “malaria stability index” (*22*). This index expresses the potential stability of transmission of malaria considering the environmental and habitat conditions favorable to its persistence, therefore quantifying an overall potential risk of transmission. We note that a high stability index does not imply the presence of malaria but rather defines its potential impact to persist if it was present. In this way, we inferred and mapped the potential stability of malaria through time. By incorporating species ecology, environmental and climatic changes, and pathogen stability (i.e., incubation period), we were thus able to map through space and time the areas where malaria had the potential of affecting humans. We then used independent reconstructions of the suitable range for humans (human niche) based on archaeological sites (*7*, *23*) to track the human expansion across the landscape (see supplementary text). Comparing these two reconstructions (potential malaria risk and human ranges) allowed us to infer and quantify the impact of malaria on human demography and dispersal.

Modeling of the variant underlying sickle cell anemia has revealed this mutation to be much older than crop domestication, most likely dating just before the Last Glacial Maximum (LGM) in the ancestors of the Bantu in West Africa (*9*). Thus, we would expect that, if *P. falciparum*–induced malaria imposed a strong burden on humans, then there would be limited overlap between human occupation and areas suitable for malaria in the past, with a progressive increase in overlap starting from West Africa during or just after the LGM (giving time for the mutation to spread enough within the population to have an effect). Our reconstructions show exactly that pattern, indicating that human occupation patterns have been substantially shaped by the presence of malaria.

## RESULTS

### Spatial distribution models of the studied *Anopheles* vectors

The use of SDMs enables us to reconstruct the realized niche of the studied vector species by linking their known occurrences to environmental and climatic variables at their locations to retrieve the potential distribution over a whole area (*21*, *24*). We focus on occurrences, rather than abundances, because current local mosquito density is heavily affected by urbanization patterns and human healthcare interventions (such as the use of insecticides). Occurrences, on the other hand, should be relatively robust to such factors as large-scale eradication of vectors is still rather limited. Anthropogenic land use provides an additional potential driver of the distribution of *Anopheles* mosquitoes (and thus of malaria). To capture the changes in local habitat due to land use change, we use leaf area index (LAI) as a quantitative proxy of the structure of vegetation in both natural and anthropogenic landscapes. LAI was estimated and adjusted for the present by combining estimates based on natural vegetation as predicted from climatic variables with the proportion of habitat converted to crops, pasture, and grazing land at any given location (see supplementary text). We did not consider human population density, as we wanted to build models that would focus on the climatic drivers of mosquito distributions to be able to project back in the past when humans were hunter-gatherers (and thus their densities were not comparable to current levels) (*25*). Because our SDM focuses on the presence over a large geographic area rather than density of mosquitos, this omission should not affect the SDM much as the presence of a species across the continent should be driven more by the climate, while it is its local abundance that will be affected by other factors such as the density of the hosts.

**Fig. 1.**
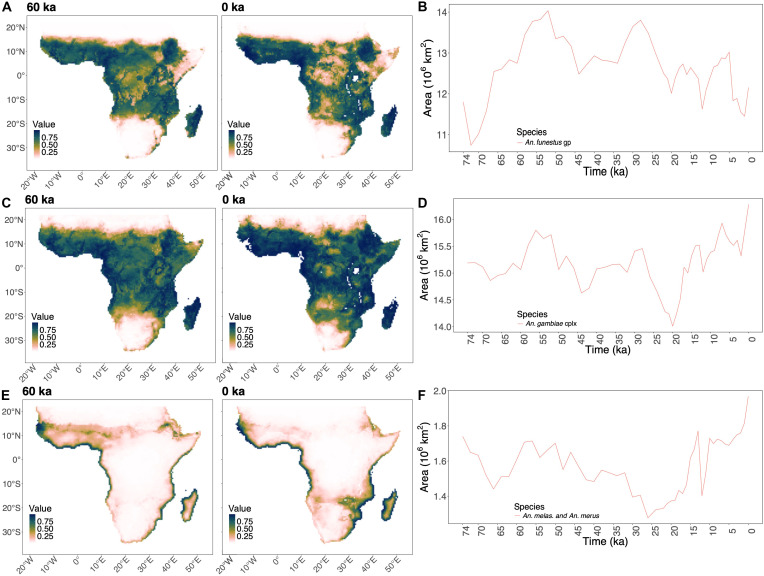
SDMs showing the changes in distribution ranges through time. (**A**) SDMs of *An. funestus* group (gp) at 60 ka and in the present (0 ka) with (**B**) range of core areas (0.95%) of distribution through time; (**C**) SDMs of *An. gambiae* complex (cplx) at 60 ka and in the present (0 ka) with (**D**) range of core areas (0.95%) of distribution through time; (**E**) SDMs of *An. melas* and *An. merus* at 60 ka and in the present (0 ka) with (**F**) range of core areas (0.95%) of distribution through time. Note that the different *y* axis in (B), (D), and (F) reflects the different extent (area) occupied by each species.

We generated SDM for three selected malaria vector groups: *Anopheles gambiae* complex, its salt-water breeding species as a separate group (*Anopheles melas* and *Anopheles merus*), and *Anopheles funestus* group (see Materials and Methods). These species represent dominant vectors identified taxonomically in historical records and, among different species of mosquito vectors, hold the most substantial impact (see Materials and Methods). Nonetheless, past environments may also have been suitable for other mosquito species with more zoophilic or opportunistic behavior. To explore the robustness of our conclusions to the choice of vectors, we repeated all our analyses considering several additional species (including some that also affect primates and cattle) in the Supplementary Materials, with no qualitative impact on our key conclusions. We combined the species observation data with environmental predictors to map habitat suitability (from low to high) across the landscape (exemplified in Fig. 1, A, C, and E) for the whole period analyzed (Fig. 1, B, D, and F). The best models were selected among the predictions in an ensemble (*26*) with a minimum threshold of 0.7 for the maximum true skill statistics (TSS). The obtained vector distributions in the present, reconstructed with these selected variables and map resolution, are coherent with presence data and previously published SDMs (see supplementary text) (*27*). This latter coherence between our SDMs, which focus on climate, and previous work, which also included human population density, validates our logic that the continental-scale geographic distribution of mosquitos is mostly driven by climate. We identified the coastal distribution for species such as *An. melas* and *An. merus* (Fig. 1, E and F) and highly suitable areas in West and Central Africa for the rest of *An. gambiae* complex species (Fig. 1, C and D).

### Potential malaria risk through time

We combined the effects of the presence of multiple mosquito species to generate a malaria stability index (*22*) representing the potential risk of malaria transmission as an infectious disease at a given location. This potential risk of *P. falciparum*–induced malaria reflects the stability of transmission (i.e., consistent transmission throughout the year) considering the favorable (ecological) conditions to malaria, conceptually equivalent to the modeling of habitat suitability of a species (e.g., the mosquitoes’ ecological niche using SDM). Thus, the malaria stability index can indicate the ecological conditions linked to high risk of malaria and, consequently, malaria’s potential range. High values of the index do not imply the presence of the disease, but rather its likely persistence if it was present (again, analogous to the suitability recovered from an SDM). In this context, current sedentary lifestyles and the presence of people do not have an impact on the potential range, but affect the incidence of malaria, increasing the actual risk of transmission. The malaria stability index, therefore, expresses the potential stability of transmission and combines the impact of each vector present based on its ecology and its contribution to the persistence of *P. falciparum* (factors that consistently and strongly affect the stability of malaria transmission). Nevertheless, it has been shown to also correlate well with the incidence of the disease (*22*), although we caution that this relationship might have been weaker in the past if other vectors were involved. We computed the malaria stability index over the past 74 ka across sub-Saharan Africa.

**Fig. 2.**
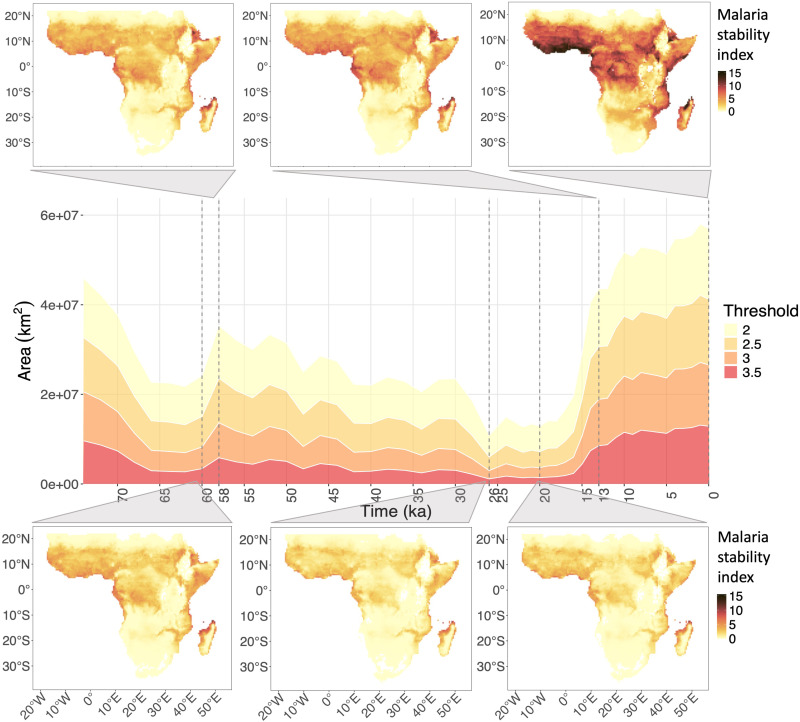
Malaria stability index through time, considering an environment affected by land use. The areas in the map (square kilometers) that have an index value above a set of arbitrary thresholds (i.e., high malaria levels) are on the *y* axis, while time [thousand years ago (ka)] is on the *x* axis. The same pattern can be seen independently from the choice of threshold. Six different time steps are highlighted: At 60 ka, 26 ka, and 20 ka we see a decrease in stability of overall malaria, while, at 58 ka, 13 ka, and present (0), we see an increase.

The pattern that emerges shows a clear increment of malaria stability over time across the landscape (Fig. 2). The first visible peak of malaria corresponded with the main “Out of Africa” expansion at about 60 to 50 ka, making it more probable that any group leaving the continent would carry the disease with it, as suggested in (*28*). Climatic conditions in Southern Asia would have been likely suitable for other mosquito vectors to sustain the infection. Then, the major peak occurred shortly after the LGM, at around 13 ka, showing that an increase in the extent of areas with high suitability for malaria was taking place well before agricultural practices began to emerge around 8 ka. This result is in line with the finding by Laval and colleagues (*9*) that selection pressure of malaria predated crop domestication, suggesting a period of possible intense contact with the disease. We note that our results do not conflict with the suggestion that sedentary lifestyles and increased population sizes had independent effects on local incidence of malaria. As we highlighted before, the obtained malaria stability index expresses the potential risk of malaria and quantifies its range, rather than the local incidence of the disease.

### Malaria impact on human range expansion

We then tested the impact of potential malaria risk on human settlement patterns by comparing the changes in our malaria stability index reconstructions across sub-Saharan Africa with independent reconstructions of the potential distribution of hunter-gatherers over the same period and area [based on (*23*), an extended version of (*7*); see supplementary text], considering a time frame from 74 to 5 ka to focus only on the hunter-gatherer population. These reconstructions of hunter-gatherers’ distribution were obtained using SDMs to reconstruct the ecological niche of humans by modeling the relationship between climatic variables and the distribution of archaeological sites (see Materials and Methods). These occurrences were based on a curated pan-African dataset of archaeological sites [based on (*7*), and expanded to 5 ka (*23*)], and allowed the niche to change through time (*29*). It is worth highlighting that this reconstruction is completely independent from our analyses on malaria, but, because it uses the same palaeoclimatic data (*30*, *31*), we can compare the two reconstructions.

**Fig. 3.**
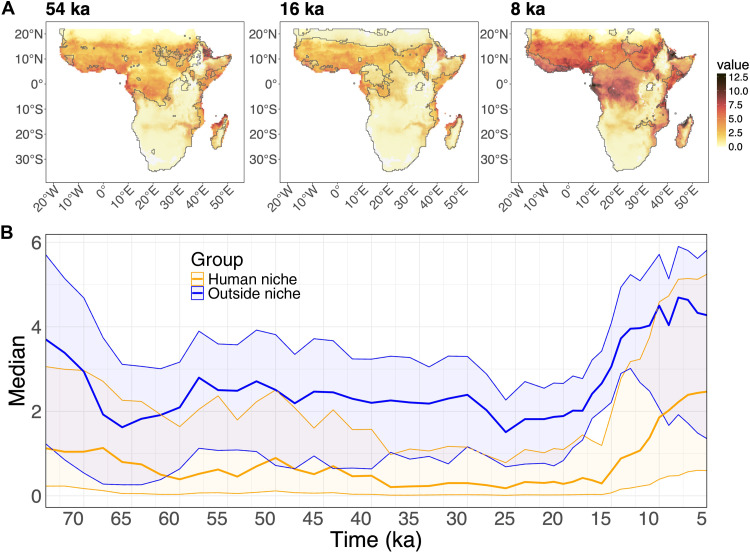
Comparing the extent of human niche and malaria stability index through time. (**A**) Extent of the human niche (outlined in black) against the map of malaria stability index at 54, 16, and 8 ka; (**B**) median of level of malaria stability index in the area of human range (dark orange line) and outside the area of human range (dark blue line), including the uncertainty (interquartile, color in transparency around the darker lines that shows median values). We can see that the level of malaria in the human niche is consistently lower than the areas avoided by humans.

The pattern that emerges reveals a negative relationship between areas of high malaria stability and suitability for *H. sapiens*. This indicates that human expansion within Africa was markedly affected by the presence of *P. falciparum*–induced malaria and that humans likely avoided areas with a high potential risk of malaria transmission through time (Fig. 3; see fig. S5 for more time steps). In Fig. 3A, the regions that are most likely inhabited by humans (core areas; see Materials and Methods) are superimposed (black outlines) onto the map of the potential risk of *P. falciparum*–induced malaria. The figure shows how areas of low stability of malaria transmission were consistently more suitable to human inhabitation. Through time, this created potential corridors for human movement and expansion as well as pockets of isolated human groups. For example, it is possible to notice that areas between the Saharan and Ethiopian habitable zones underwent cycles of disconnection (e.g., at 54 and again at 8 ka in Fig. 3) and connection (e.g., at 16 ka in Fig. 3).

To further explore the pattern, we estimated the level of malaria stability index (median and interquartile) across the landscape, dividing the map in (core) “human niche” (based on the core area that would include 95% of archaeological sites) versus “outside the niche.” We can see that the level of malaria stability index was much lower in the areas suitable for humans than in the areas outside the human range, and this difference persists through time (Fig. 3B). The human niche was characterized by consistently low values (Fig. 3B and, for example, 54 ka in Fig. 3A) from 74 ka until around 13 ka. It is also possible to notice that, from around 14 to 13 ka, the overlap between malarious areas and the periphery of human ranges increased, especially in West Africa, meaning that humans were possibly venturing in areas with higher malaria risk they were not reaching before. By 10 ka, we can see increased overlap, reaching the highest values especially in West Africa (see 8 ka in Fig. 3A) where we expect the initial spread of the resistance mutation sickle cell anemia.

## DISCUSSION

Our results show that the potential risk of malaria transmission shaped the spatial organization of human groups at least over the past 74 ka, structuring populations into different regions of sub-Saharan Africa and creating barriers that reduced contact between some local populations from others and possibly contributed to the population structure revealed by genetic analyses (*32*, *33*). The overlap between high malaria risk areas and human ranges becomes more evident after 15 to 14 ka in West Africa. This pattern matches our expectation given the dating of the origin of the sickle cell mutation in the ancestors of the Bantu in West Africa (*9*), providing an indirect validation to our reconstructions. This pattern is found both with SDMs based only on the three main groups of contemporary vectors as well as SDMs for individual vectors including less important species (see supplementary text), suggesting that these three groups likely played an important role from the early stage of the interaction between humans and malaria (although it does not exclude an important role of other species that might now be less prominent).

Furthermore, our results show that malaria was already at an extremely high level around 13 ka, before the suggested advent of agropastoral lifestyle around 8 to 7 ka. Our results, therefore, indicate that, due to climate conditions, malaria was already a potential major driver of selection before crop domestication. These results also caution against making assumptions regarding links between Neolithization and the origins of several infectious diseases [e.g., (*34*–*36*)]. How and why pathogens emerged and spread and affected human populations beyond health is clearly a critical dimension of the human past that is still largely unexplored.

Until now, the lack of availability of ancient genomes for direct evidence and refined dating of malaria in the Pleistocene has been thought to present a challenge by limiting studies to historical and modern data from *Plasmodium* (*18*, *37*) and from living people (*9*). This study provides proof of concept that we can explore disease burden in the earliest periods of human prehistory without direct evidence of human-pathogen interactions. It is also instructive that climate alone can potentially determine disease presence without complex anthropogenic habitat intervention or major changes in subsistence economy.

This could be the case for many diseases. Our methods and approach provide a way to understand disease burden in the past and how this may have affected the spatial organization of human groups, their patterns of dispersal, and their degree of contact/isolation, representing an additional and innovative dimension of knowledge for understanding the dynamics shaping the formation of our species.

## MATERIALS AND METHODS

### *Anopheles* species

We studied three *Anopheles* mosquito groups that are major vectors of malaria: *An. gambiae* complex, *An. melas* and *An. merus* (considered as an independent group being a subset of *An. gambiae* complex with specific saltwater-breeding habits), and *An. funestus* group. It is important to note that differentiation of sibling species within the *gambiae* complex was not possible until the 1990s or earlier through cross-mating (e.g., differences between *An. melas* and *An. gambiae* or between *An. gambiae* and *Anopheles arabiensis* in the 1960s). Different *Anopheles* species show different breeding periods, feeding patterns, survival rates, and, therefore, different distribution and competence (*22*, *27*), strongly determined by the climate and environment (*38*). It is also important to consider the interactions between vector species: When a habitat is invaded, other vectors may be displaced. A competent vector (i.e., a vector that is anthropophilic, more abundant than other *Anopheles* species and that frequently contains sporozoites) is defined as “dominant” (or primary) (*22*). Kiszewski co-workers’ model (2004) recognizes a different occurrence of dominant vectors during different seasons within a region, considering only the contribution of one dominant vector as the primary determinant of endemicity in the region while ignoring secondary vectors (*22*). This information and expert contribution guided this study in the choice of species to include in the model, creating a curated dataset that ensured correct taxonomic identification and based on the most comprehensive and complete historical surveys.

Among the *Anopheles* species that are known to inhabit sub-Saharan Africa, we selected dominant vectors and relevant complexes to cover different geographical distributions, biome and host preferences [expert information; (*27*, *39*)]. See table S1 for the list of species and sibling groups covered. Our reconstructions are thus in line with recent work (*27*, *39*), reconstructing the expected niche in the present (see the Supplementary Materials for tested niche overlap).

Our approach makes the important assumption that the niche of the various mosquito species considered in this work has not changed substantially over the past 74 thousand years, because we use a contemporary SDM to project back in the past. While mosquitoes have short generation times and their evolutionary responsiveness to human interventions [e.g., resistance to dichlorodiphenyltrichloroethane (DDT)] contributes to their success, there is no recent radiation that would suggest major changes in niche as a response to humans [overall plasmodium-carrying mosquitoes possibly emerged about 46 million years ago (Ma) (*40*); the split between *An. gambiae* and *Anopheles coluzzii* is dated to about 2 Ma (*41*)]. Furthermore, the correspondence between the inferred origin of sickle cell anemia and the timing and location of increase in overlap between the reconstructed human niche and malaria risk provides an indirect validation of the plausibility of our reconstructed ranges of the vectors.

### Summary of species distribution map generation

#### Environmental and climatic data

The *Anopheles* mosquito vector species are influenced by climate, which is an important factor for the vector’s spatial distribution range (*42*). Individual sibling complexes show different relationships with climatic factors, especially temperature, humidity/wetness (*27*), and precipitation (*43*). Temperature also affects the duration of sporogony of the parasite in the mosquito (*44*).

The area of study was limited to sub-Saharan Africa including Madagascar. To reconstruct the environment map, we used climate simulation outputs based on HadCM3 from Beyer *et al.* (*30*) [via pastclim v 1.2; (*45*)]. These simulations are provided downscaled to 0.5° [see (*30*) for details], as a set of rasters from the present with intervals of 1000 years up to 22 ka and then of 2000 years up to 74 ka (*30*). This dataset includes 19 variables: 16 BioClim variables excluding BIO2, BIO3 and BIO15 [in (*30*)], LAI, net primary productivity (NPP), and topography (rugosity, a measure of the standard deviation in altitude within each cell). Comparison to paleoproxies has shown these climate simulations to capture well the prevailing climate patterns in Africa over the past 20 thousand years (*46*).

To reconstruct both a natural, pristine environment and a modified environment (i.e., affected by agriculture or pastoralism, here defined as “land use”), we adapted land use variables from the History Database of the Global Environment [HYDE version 3.2; (*47*)]. We considered cropland, grazing land, and pastures from HYDE version 3.2 to reflect the type of vegetation in Africa from 10 ka until the present. To define an “open” and “closed” type of vegetation and, therefore, reflecting the type of environments favored by mosquitoes, the LAI was used as a proxy. The cropland and pasture variables were converted to LAI [with LAI of 1.7 for both (*24*, *48*)] creating the variable “land use.” Grazing variable from HYDE version 3.2 was used as a proxy for cattle. Additionally, the distance from the sea (in kilometers) was included as a variable for coastal vector species like *An. merus* and *An. melas*.

### Species distribution models

#### Data cleaning and thinning

Data points of presences were obtained from two large, curated databases that synthesize most of previous surveys from (*27*) and (*39*), and additional data were sought in regions that appeared undersampled [specifically *An. funestus* sites curated by experts and based on (*49*) and (*39*)]. This approach aimed to minimize the inherent biases associated with occurrence data (e.g., their opportunistic nature, leading to potentially uneven sampling effort). Using the R package tidysdm (*50*), the data points were thinned, first keeping one observation per cell and then ensuring that there was a minimum distance of 70 km between each observation.

#### Model fitting

SDMs were performed using the R package tidysdm (*50*). The thinned dataset was used as presences (*27*, *39*, *49*). Three times the number of presences was drawn as pseudo-absences keeping a minimum distance of 150 km from the presences, and this procedure was repeated 20 times, creating 20 independent sets of randomly selected pseudo-absences to account for uncertainty in their spatial placement and mitigating potential biases. The analyses were conducted at a relatively coarse spatial resolution (0.5° by 0.5°, corresponding to around 55 km at the equator), ensuring that each species would be potentially scored as present even in areas with limited sampling.

The variables of interest were chosen considering the proportional overlap of presences’ and pseudo-absences’ distributions over the variable space [tidysdm::dist_pres_vs_bg()], where high distance between the two distributions is indicative of nonrandom use of the area by the species (table S3). Climatic and environmental variables were further selected to better capture the difference between Western and Eastern Africa at the current map resolution (0.5° by 0.5°), leaving, for each species group, a combination including some of the following variables: BIO5, BIO6, BIO4, BIO8, BIO16, BIO18, BIO19, NPP, rugosity, LAI, grazing, and distance from the sea. Then, these selected variables were pruned for collinearity [i.e., greatest mean correlation, with a cutoff correlation coefficient (*r*) of 0.8].

We built an ensemble of models for each set of presences and absences using four different algorithms: generalized linear models, random forest, generalized boosting method, and maximum entropy (MaxEnt) (*51*). Using a workflow in tidysdm, we tuned the models with a spatial block cross-validation scheme (*52*), with an 80:20 split (i.e., four-fifth of the splits are used for calibrating the model and the remaining one-fifth for evaluation) by creating five folds. For each algorithm, we explored 20 combinations of the hyperparameters [based on (*53*)], using the maximum TSS as a metric to choose the best combination of hyperparameters. Only models with a TSS larger than or equal to 0.7 were retained. The resulting 20 ensembles were further combined into a “repeated ensemble” by taking their median predictions using tidysdm.

The predictions for the present were in line with previous continental-level SDM, which included variables describing human densities (*27*), confirming out choice of variables, including the impact of land use change (see Supplementary Text and fig. S2, A to C).

We then projected the obtained model into the past up to 74 ka (fig. S3 showing some time steps for some species). The contribution of each variable to the models was also explored (table S3).

### Epidemiological information: The malaria stability index

Once the niche of dominant species of mosquitoes was identified through time, we calculated an index for the stability of malaria transmission at each time step, based on (*22*). This index represents a potential risk of malaria transmission (i.e., its potential impact if it was present), expressing the potential stability of transmission of malaria considering the environmental and habitat conditions favorable to its presence. The index includes *P. falciparum* incubation period, vector’s biting activity (i.e., proportion of bites on humans by the dominant vectors), daily survival rate of the vectors, and monthly temperature

$$\sum_{m=1}^{12}a_{i,m}^{2}p_{i,m}^{E}/-\text{ln}\left(p_{i,m}\right)$$

where E=111/Temperature−16°C for *P. falciparum*.

Last, we multiplied the median probability of vectors’ incidences from the obtained SDMs by the malaria stability index to find the areas with the highest incidence rate. For a detailed explanation of the methods and interpretation of the malaria stability index, see the Supplementary Materials.

### Correlating the human niche to the malaria stability index

Independent reconstructions of the human niche were obtained from (*7*) and (*23*) (see the Supplementary Materials). The reconstructions were based on archaeological sites across Africa, with dating starting from 120 ka, considering five climatic and environmental variables (*54*): LAI, temperature annual range (BIO7), mean temperature of the wettest quarter (BIO8), mean temperature of warmest quarter (BIO10), and precipitation of wettest quarter (BIO16). The niche area was divided into core areas (the smaller area that includes the 90% of the presences) and extended areas (covering 95 and 99%) by (*7*). We chose a level of 95% as the range of humans across the landscape [see (*7*), where “core area” is instead defined as an area encompassing 90% of presences].

First, we checked the spatial overlay of the human core area against the maps of malaria stability index extent across time (see Fig. 3A). Then, we compared the median level of the malaria stability index in the areas identified as core areas for humans against the median level of the malaria stability index in the areas outside the human range, i.e., areas identified as not suitable for human groups (considering upper and lower quantiles of 0.25 and 0.75). Desert areas (unsuitable for both humans and malaria vectors) were still included in this analysis.

## Supplementary Materials

### The PDF file includes:

Supplementary Text Figs. S1 to S7 Tables S1 and S2 Legends for tables S3 to S6 References
