# Supplementary material for: Malaria shaped human spatial organization for the past 74 thousand years
Source: Sci Adv. 2026 Apr 22;12(17):eaea2316. doi: 10.1126/sciadv.aea2316 (PMC13101881; doi:10.1126/sciadv.aea2316)
Supplement: Supplementary file 1 — Supplementary Text Figs. S1 to S7 Tables S1 and S2 Legends for tables S3 to S6 References [file sciadv.aea2316_sm.pdf]

Science Advances
AAAS

Supplementary Materials for

Malaria shaped human spatial organization for the past 74 thousand years

Margherita Colucci *et al.*

Corresponding author: Margherita Colucci, colucci@gea.mpg.de; Andrea Manica, am315@cam.ac.uk; Eleanor M. L. Scerri, scerri@gea.mpg.de

Sci. Adv. **12**, eaea2316 (2026)
DOI: 10.1126/sciadv.aea2316

The PDF file includes:

Supplementary Text
Figs. S1 to S7
Tables S1 and S2
Legends for tables S3 to S6
References

Other Supplementary Material for this manuscript includes the following:

Tables S3 to S6

Supplementary Text

1. Human Origins

In the last few years, there has been a profound reconsideration of the character of human evolution, and the origin of our species *Homo sapiens* in Africa. Rather than, as the word "origin" suggests, emerging from a single centre of endemism, the set of cognitive and behavioural traits that define our species emerged through an evolutionary process that played out across much of the African continent. Over time, and through the genetic exchanges between diverse sets of local populations living in different regions of Africa, the set of physical and behavioural features that defines *H. sapiens* began to emerge. This pattern is evidence both through fossils and material culture. Fossils bearing modern traits appear in different African regions from around 300 thousand years ago (ka) (*3, 51*), with some archaic features lasting until relatively recently (*52*). Similarly, Middle Stone Age (MSA) material culture, which marks a fundamental re-organisation of technological conceptions, appears with some of the earliest *Homo sapiens* fossils around 300 ka (*51, 53*). Thought to be the earliest manifestations of modern cognition, the MSA also appears in many different African regions at around the same time (*3, 54*).

Most recently, studies using genetic data from contemporary populations have supported the view that humans are one species with several African roots, bearing out a range of prescient model-based and theoretical studies (*3, 55*). Ragsdale and colleagues (2023) (*2*) presented a study showing that multiple stem populations contributed to the emergence of our species. This early population structure is therefore key to understanding the character of modern human origins. However, the mechanisms driving population structure are not well understood. Currently, all that seems clear is that the earliest members of our species lived within a structured metapopulation, whose dynamics shifted over time, with modern-day population structure originating before 300 ka (*10, 32, 56*). It seems likely that some of the same mechanisms drove this shifting and dynamic population structure. Chief among candidate mechanisms is climate. Africa's fractured palaeoclimates have been well documented, particularly from the Last Interglacial (~130 ka) (*57-60*). The ebb and flow of the Sahara Desert (*61*), "mega" droughts in Central Africa (*62*), moisture "see-saws" between eastern and western Africa (*59*) among other examples, all contributed to differential climate dynamics across Africa. As a result, they are also likely to have impacted the dynamics of human habitation, dispersal, and encounters between local groups that underpin genetic exchanges. They may also underpin similar patterns of sub-structure observed in the genetics of other pan-African mammal species (*63*).

Beyond this, geographic distances, and potentially, culture drove some degree of population structure, as they do today (*54*). However, disease has historically not been considered as a factor driving habitat choice and human demography in the African Pleistocene, despite the major influence on human populations' health.

2. The study of past diseases: malaria as a case study

Diseases have shaped human behaviour through time, often in extreme ways, from past epidemics (e.g., plague), to present pandemics (e.g., COVID-19). However, our ability to analyse and quantify the actual disease burden has been limited, especially when considering longer times-scales. For example, recent studies revealed the complex history of hepatitis B virus

(HBV), revealing it presence in Neolithic, Bronze Age, as well as Iron Age (*64, 65*) and transmission between ancestors of European and Native American populations around 15-13 ka (*64*) highlighting the ancient origin of this disease (*7*) and suggesting the potential burden on the population already in the late Pleistocene. However, no direct evidence of prevalence or effects is available on this ancient virus.

Some vital information can be obtained by the application of indirect methods. For example, genetic analysis of modern (and ancient) human samples enables us to indirectly explore the impact of pathogens on human genomes (and populations). This can be done by examining the selective pressure imposed by the disease as a result of host-pathogens encounters and pathogenic load (*8, 66*). An example is the impact of malaria on human populations: due to its considerable burden on human health, several mutations that offer a level of protection emerged alongside it, making this disease ideal for our study.

Due to its high morbidity and mortality, with around 263 million cases globally (*17*) malaria continues to be a major public health concern. Malaria is caused by *Plasmodium* parasites, of which five types exist: *Plasmodium falciparum*, *Plasmodium vivax*, *Plasmodium malariae*, *Plasmodium ovale*, and the zoonotic/simian *Plasmodium knowlesi*. Among these, *P. falciparum* is the most virulent of human malaria parasites (*67*). The extensive distribution of these parasites and the burden of the disease placed one of the highest selective pressures on the human genome, leading to the emergence of resistance mutation (i.e., mutations that offer a level of protection), such as sickle cell anaemia (*68*), α and β-globin mutations in thalassemia, Duffy blood group variants (*69*), tumour necrosis factor (TNF), and glucose-6-phosphate dehydrogenase (G6PD) (*70*).

In this paper, we focus on *P. falciparum* and haemoglobin β^s sickle mutation, which has been reported with high frequency in areas of high malaria density, particularly in sub-Saharan Africa (*71, 72*). While the homozygous form of this mutation can lead to sickle-cell disease and potentially death, the heterozygous form offers higher protection against malaria (*73, 74*). Because of its distribution and malaria-resistance effect, the emergence of β^s sickle mutation in humans has been studied to better understand the history of malaria itself.

Theories on the origin of malaria (*Plasmodium falciparum*)

Despite sustained research attention, malaria's epidemiological and evolutionary past remains only partially understood. There are various hypotheses, considering both a multicentric model, with five independent occurrences spanning a time range of a few thousand years, and a unicentric model, with an older, single occurrence (*16*). These hypotheses consider central (*68, 75*) and west Africa as points of origin (*71, 76, 77*). These areas are further explored in this paper.

Traditionally, malaria is linked to the emergence of slash-and-burn agriculture in the past 10-8 ka (*68*). Authors have highlighted fundamental factors such as a more favourable climate and environment in Africa (about 12-7 ka) for plant and animal domestication (about 10-8 ka in the Sahara and northeast Africa), for human settlements as well as malaria vector species (*70*). Considering the emergence of the original sickle haplotype, a time frame predating the Bantu

expansions, around 7300 ya, was proposed (*16*). However, there are various lines of evidence that nonetheless backtrack its presence.
Considering the age of *P. falciparum*, it was demonstrated that the plasmodium existed before agriculture (90% credibility intervals), and it was linked to the movements of the human host from sub-Saharan Africa, following humans during an expansion out of Africa around 60–50 ka ago (*28*).

Another line of evidence comes in the form of genetic selective pressure due to malaria disease burden. Laval and colleagues (*8*) tracked sickle cell mutation to date the start of selective pressure by the most virulent form of malaria, transmitted by *P. falciparum*. Focusing on rainforest hunter-gatherers and agriculturalist groups, they identified 25-22 ka as the start of sickle cell anaemia distribution/the rise of the sickle cell mutation. This study highlights the ancient origin of malaria, breaking the usual narrative that links the emergence of diseases to the emergence of crop domestication (e.g., (*34-36*)). The frequencies of sickle cell used in (*8*) are inferred from modern individuals from publicly available resources of global human genetic diversity (1000 Genomes Project (*78*) and HapMap (*79*)). However, it also offers a wide range of confidence intervals on dates: the question on when malaria, as a major disease developed, is still open. With our methods, we propose a reconstruction of the potential risk of malaria in sub-Saharan Africa since the late Pleistocene. Our reconstructions corroborate Laval *et al.* (*8*) findings, and allow us to infer how human behaviour is heavily shaped by the presence of a disease.
The ability to investigate the link between disease and broader patterns of human behaviour, land use, and demography is therefore of considerable importance. Such relationships have been explored for more recent timescales using a variety of approaches, including studies suggesting that settlement clustering may have been intended to minimise the risk of virulent epidemics (*80*), analyses of the impact of malaria on settlements, agriculture, and demography in historical contexts such as Ancient Rome and during the Imperial period (100 CE; (*81, 82*)), and assessment of how other vector-borne diseases influenced access to and use of land (*81*). Our study highlights the importance of including disease in the broader picture and, for the first time, proposes a quantitative approach to investigate disease impact on human settlement patterns and behaviour in the deep human hunter-gatherer past.

<u>The focus on malaria vectors</u>
Understanding malaria by studying its vectors is crucial (*48, 83*). Malaria is transmitted by mosquito species in the *Anopheles* genus, which includes around 400 species, with about 70 of relevance for human health (*43*). The diversity shown among *Anopheles* species highlights the importance of identifying and mapping the most impactful malaria vectors.

Particularly in Africa, it is possible to observe considerable variability in the geographic distribution of sibling species. For instance, *An. gambiae* has a wide range, expanding particularly in West Africa, while other species, like *An. melas* and *An. merus*, favour coastal areas (i.e., saltwater breading), but can be found further inland too (*48*). *An. funestus* has extended ranges to the point of being described as “ubiquitous” (*48*). Therefore, a better understanding of the ecological factors that favour anopheline species to implement the use of ecology-based models is necessary for understanding and predicting malaria transmission and distribution (*84*).

The question of environmental and anthropogenic changes

As described in the previous section, plasmodium survival and malaria vector distribution are profoundly affected by favourable environmental and climatic conditions. Currently, malaria incidence is strongly determined by population density, with substantial impact from climatic and environmental factors such as temperature and water availability. Also, the increase of open habitat, such as the conversion of natural land cover to cultivated land, favours an increase in the range of the mosquito vector (*85*). In this paper, we considered the environmental conditions prior to possible human expansion and intervention, and the subsequent changes through time from the Late Pleistocene. Gosling *et al.* (*60*) highlight that the available records (from western and central Africa, Lake Bosumtwi and Lake Bambili) show marked vegetation change (from forest to savannah, with a decline of tree line) around 300–50 ka (*60*). Despite being generally associated with this type of open landscape, humans and evidence of human activity can be found in a variety of different environments, also attested by a change in technology and tools to possibly accommodate new environmental challenges (*60*).

3. Methods

To explore the extent of malaria spread in the past and its influence on human population ranges, we reconstructed the environmental niche of its sub-Saharan mosquito vectors, the inferred the potential risk of malaria (i.e., malaria stability index), and we compared the potential risk of malaria against the potential range of *Homo sapiens.*

The workflow to reconstruct past malaria distribution includes three main steps (see fig. S1):

- Species Distribution Models (SDMs) based on (A) observed presences of mosquito vectors and, independently, on archaeological sites associated with *H. sapiens* presence across time and space, reconstructing their distribution, considering climate and environmental models (steps B-C)
- Calculating the malaria stability index based on climatic and epidemiological information on the plasmodium and the vectors (step D);
- Then, comparing the obtained independent reconstruction (SDMs, step E) of the human habitat range across Africa against the obtained range of potential risk of malaria.

These steps will be described in detail in the next paragraphs, starting with the description of data used.

Anopheles species

A competent vector (i.e., a vector that is anthropophilic, more abundant than other anophelines species and that regularly harbours sporozoites) is defined as "dominant" (or primary) (*22*). Kiszewski co-workers' model (2004) (*22*) recognises occurrences of different dominant vectors during different seasons within a region, considering only the contribution of one dominant vector as the most influential factor in determining endemicity in the region, while ignoring secondary vectors. This information guided this study in the choice of species to include in the model. We studied three *Anopheles* mosquito groups that are dominant vectors of *P. falciparum*: *An. gambiae* complex, *An. melas, An. merus*, and *An. funestus* group. Different *Anopheles* species show different breeding periods, feeding patterns, survival rates, and, therefore, different distribution and competence (*22, 27*), strongly determined by the climate and environment (*38*).

Their occurrence and abundance are also influenced by interactions between vector species: when a habitat is invaded, other vectors may be displaced. This is the case for *An. funestus* and *An. gambiae* complex species, as they interact across the season maintaining a certain level of malaria yearly (*22*).

Among the *Anopheles* species that are known to inhabit sub-Saharan Africa, we selected dominant vectors and relevant sibling complexes to cover different geographical distributions and biomes (expert information; (*27, 39*)). These species have been demonstrated to consistently transmit malaria in the studied area (*22*). *An. gambiae, An. coluzzii,* and *An. arabiensis* are three vectors (here included in the *An. gambiae* species complex as in historical records) that show the most extensive ranges across sub-Saharan Africa, with some possible natural barriers (e.g., *An. gambiae* is limited by Rift Valley complex (*86, 87*), *An. coluzzii* by the Congo Basin tropical rainforest, and *An. arabiensis* by the Indian Ocean) hindering possible migration (*87*). See table S1 for the list of species and sibling groups covered.

Data sources

Presences were obtained from (*27, 48*) and expert information, creating a curated dataset based on taxonomic identification and able to cover a wide range of species-specific habitats.
The observations were limited to sub-Saharan Africa, including Madagascar, but excluding smaller islands such as Comoros and Mayotte.

Summary of species distribution map generation

Environment and climate reconstructions

The *Anopheles* mosquito vectors' spatial distribution ranges are influenced by climate (*88*). Individual vectors show different relationships with climatic factors, especially temperature and humidity/wetness (*27*) and precipitation (*43*). Wet and dry cycles maintain levels of malaria transmission through interactions between dry-season and wet-season vectors (*22*). Stable levels of heath favours stable presence of mosquitoes (e.g., savannah areas in west and central Africa) (*22*). Temperature also affects the duration of sporogony of the parasite in the mosquito (*44*). Topography is also considered an important factor alongside climate and anthropogenic intervention (*85*).

The area of study was limited to sub-Saharan Africa as a core region of early human prehistory. To reconstruct the environment map, we used a dataset at a native resolution of 0.5° x 0.5° (corresponding to around 55 km at the equator) and time step reconstructions from the present with intervals of 1000 years up to 22 ka, then of 2000 years up to 74 ka (*30*). This dataset includes 19 bioclimatic variables: all the BioClim variables (excluding BIO15) (*89*), Leaf Area Index (LAI, a measure of canopy foliage used as a proxy for vegetation), Net Primary productivity (npp) and rugosity (a measure of the standard deviation in altitude within a certain area to reflect topography).

To reconstruct both a natural, "pristine" environment and a "modified" environment (i.e., impacted by conversion of land to crop, pasture and grazing land with opening of the vegetation, here described as "land use" and "land use with grazing") we adapted land use variables (i.e., cropland, pasture, grazing) from the History Database of the Global Environment (HYDE version 3.2, (*46*)). We considered cropland, grazing land, and pastures from HYDE v. 3.2 to

reflect the type of vegetation in Africa from 10 ka until the present. The effect of land use was modelled by using the Leaf Area Index (LAI) thus defining an "open" and "closed" type of vegetation and, therefore, as proxy for the type of environments favoured by mosquitoes. The cropland and pasture variables were converted to LAI (with LAI of 1.7 for both) creating the variable "land use" considering the proportion of LAI corresponding to an agricultural environment in Africa (which we considered to be closer to a grassland landscape as calculated in (*24, 47*) rather than intensive cropland from world-wide measurements (*24, 47*)). Grazing variable from HYDE v. 3.2 was used as a proxy for cattle. Additionally, the distance from the sea (in km) was included as a variable for coastal vector species like *An. merus* and *An. melas*.

Species Distribution Models

The use of Species Distribution Models (SDM) enables to reconstruct the realised niche of the studied species by mapping its occurrences at known locations to the environmental variables of its habitat (*21, 24*). This produces a model of the possible distribution of the species across the landscape informed by the most suitable climatic conditions.

Model fitting

SDMs were performed using the R package *tidysdm* (*49*). The observations were thinned to have one occurrence per cell, controlling for spatial autocorrelation, sampling bias, and this thinned data set was used as presences (*27, 48*). Explicit absence-data is difficult to obtain, and, even when available, it is biased by sampling effort (trapping intensity, proximity to urban setting, etc.). This is a common problem in ecological sampling (*90*), and pseudo-absences are routinely used (e.g., (*91*)). Three times the number of presences was drawn as pseudo-absences keeping a minimum distance of 150 km from the presences, and this procedure was repeated 20 times creating 20 independent sets of randomly selected pseudo-absences to account for uncertainty in their spatial placement and mitigating potential biases.

Climatic and environmental variables were selected to better capture the difference between West-East Africa at the current map resolution and to reveal a difference in distribution between presences' and pseudo-absences' distributions over the variable space (*49*), leaving BIO5, BIO6, BIO4, BIO8, BIO16, BIO18, BIO19, NPP, rugosity, LAI, grazing, and sea distance. Then, as environmental variables often show collinearity (which can affect models such as generalised linear models), these selected variables were pruned for collinearity (i.e., greatest mean correlation, with a cutoff r of 0.8).

We built an ensemble of models for each set of presences and absences using four different algorithms: generalised linear models (GLMs), random forest, generalised boosting method (GBM), and maxent (*100*). Using a workflow in *tidysdm*, we tuned the models with a spatial block cross-validation scheme (*49*), with an 80:20 split (i.e., 4/5 of the splits are used for calibrating the model and the remaining 1/5 for evaluation) by creating 5 folds. For each algorithm, we explored 20 combinations of the hyperparameters (based on (*56*)), using the Maximum True Skill Statistics (TSS) as a metric to choose the best combination of hyperparameters. Only models with a TSS larger or equal than 0.7 were retained. The resulting 20 ensembles were further combined into a "repeated ensemble" by taking their median predictions using *tidysdm*.

The predictions for the present were in line with previous continental-level SDM which included variables describing human densities. Specifically we compared the results (SDMs) of (*27*) with ours (see Fig. S2a-c), calculating the niche overlap between the two sets of SDMs projected for contemporary climate. Overall, we obtained high levels of coherence between the two sets, with Schoener's D of 0.836 and the inverse I of Hellinger's distance of 0.831 for the *gambiae* complex; a Schoener's D of 0.726 and the inverse I of Hellinger's distance of 0.731 for the *funestus* group; and Schoener's D of 0.602 and the inverse I of Hellinger's distance of 0.621 for *An. melas* and *An. merus*. These results confirm that our approach focussing only on the bioclimatic variables is able to capture the main patterns of the distributions of these 3 groups of vectors.

We then projected the obtained model into the past up to 74 ka (figure S3 showing three time steps for each species). The contribution of each variable to the models was also explored (table S3). Finally, we applied Multivariate Environmental Similarity Surfaces (MESS), which compare the bioclimatic conditions used to train the model to those of a given time step for which we want to project it. For all time steps, we found that present day conditions over the African continent cover most of the extremes that were found in past time steps, meaning that the model was mostly interpolating, rather than extrapolating outside its training conditions, when projecting into the past (Fig. S4 a-c).

Epidemiological information: the malaria stability index

The interplay between various climatic and epidemiological factors determines the relationship between vector and disease and the consequent variations in malaria transmission intensity. Therefore, we can consider the effects of multiple vectors on the potential risk of malaria (i.e., "stability" of malaria) by calculating a malaria stability index for each species. This can tell us what are the ecological conditions linked to high risk of malaria and, consequently, potential malaria's ranges.

Kiszewski and coworkers (*22*) suggest the contribution of mosquitoes as an objective measure of malaria transmission stability, which can be estimated through a spatial index. This index takes into account the characteristics of the vectors that are influenced by climate and that have an impact on vectorial capacity, such as mosquito survival rate, which affects the stability of transmission throughout the year, seasonal temperature and precipitation, which affects both mosquitoes and parasite life-cycle as well as extrinsic *P. falciparum* incubation duration.

Therefore, once the niche of dominant species of mosquitoes was identified through time, we calculated an index quantifying the potential risk of malaria at each time step, based on Kiszweski *et al.* (*22*). The index includes across the months (*m*) the *P. falciparum* incubation period (*E*), proportion of biting people of the chosen dominant vectors (*a*), and daily survival rate (*p*) of the vectors:

$$\sum_{m=1}^{12} a_{i,m}^{2} p_{i,m}^{E} / -\ln(p_{i,m})$$

where $E = 111/\text{Temperature} - 16°\text{C}$ for *P. falciparum*.

Kiszewski *et al.* (*22*) report the human biting index (hbi) for various species that inform the proportion of biting people used in the equation.

To obtain the index, we firstly iterated through each time step (from 74 ka to present) obtaining the monthly temperature and precipitation variables. Each species SDM is considered separately, and we started by masking areas on the climate map where the species is not present to reduce computation over the areas that are excluded from analysis. Then the SDMs were filtered to exclude areas (or cells on the map) where the monthly temperature is under 15°C, and the precipitation of the previous month was lower than 10 mm as it is not viable for larvae. Then we can compute E (length of extrinsic incubation period in days) for the remaining cells. The maps for each month need to be multiplied for the SDM probabilities and then, to get a yearly measure, we sum the values across months. Finally, we take the maximum index value per cell across all species to calculate an overall stability index per year considering all vectors and, therefore, to find the areas with the highest risk rate.

In this way, the malaria stability index provides an indirect measure of the combined effects of the presence of multiple mosquito species at a given location on the potential risk of malaria as an infectious disease. Based on suitable climatic and environmental conditions and focusing on stability and transmissibility, this index expresses the potential stability of transmission of malaria (*22*), therefore, quantifying an overall potential risk of transmission. We note that a high stability index does not imply the presence of malaria, but rather defines its potential impact if it was present.

<u>Correlating the human niche to the malaria stability index</u>

<u>Summary of species distribution map generation for *H. sapiens*</u>
Building on (*9*), we aimed to reconstruct the distribution of hunter-gatherer populations in Africa from 300 ka until the present. By modelling humans' evolutionary dynamics through a time-dependent SDM based on archaeological sites across the continent, we generated reconstructions of the human niche. Further details on methods can be found in (*23*).

<u>African hunter-gatherer archaeological sites</u>
The dataset included a total of 1242 sites from 120,000 cal.BP to 495 cal.BP (*9, 92*) to form a curated dataset that would reflect the presence of hunter-gatherers in Africa. While the separation between hunter-gatherers and early farmers is often blurred, particularly given earlier assumptions that the adoption of agriculture necessarily implied complete sedentism and the abandonment of hunting and gathering, this ambiguity typically operates in one direction. Hunter-gatherers who adopt crop cultivation are typically classified as farmers, whereas farming populations that continue to practice some hunting and gathering remain classified as farmers. Accordingly, the present study focuses on hunter-gatherer populations and omits sites with clear evidence of crop cultivation and other elements of farming lifestyles, as their inclusion would otherwise conflate populations with fundamentally different settlement constraints, thus obscuring the behavioural signal of disease-risk avoidance that this study seeks to detect. Therefore, only sites that were older than 8 ka and had no evidence of cultivation, pottery, or metal use (*88, 92, 93*) were included and filtered to obtain data points with published coordinates, radiometric dates, and an age error range less than or equal to 20 ky (see (*9*) for more information on the quality control steps). If 14C uncalibrated data were present, calibration

was applied using the *rcarbon* R package (*94*) considering northern hemisphere archaeological localities (basic IntCal20 (*95*)) and southern hemisphere archaeological localities (basic SHCal20 calibration). Instead, the calibrated 14C ages were used to calculate the mean age (1σ error range). When an archaeological layer had both 14C and other dating methods, the 14C mean age and error were combined with those from the other methods to determine the final mean age for the models.

<u>Environmental and climatic data</u>
An SDM based on the curated dataset of archaeological sites was performed. The paleoclimatic reconstructions of interest (*50*) were accessed through the R package pastclim (*96*), including 17 BIOCLIM variables (excluding BIO2 and BIO3, as based only on daily summaries), net primary productivity (NPP) and leaf area index (LAI). The final model included five variables: leaf area index (LAI), temperature annual range (BIO7), mean temperature of the wettest quarter (BIO8), mean temperature of warmest quarter (BIO10), and precipitation of wettest quarter (BIO16). These variables were selected for their informativeness when comparing the habitat suitability for humans distribution in the *H. sapiens* presences against the distribution of 10000 randomly sampled points in the whole area (*9*).

<u>Model fitting</u>
Using archaeological sites as a proxy for past inhabitation poses a risk of sampling biases (e.g. due to geographically uneven effort, preservation issues, or differences in the application of radiometric dating techniques) creating artefacts in the reconstructed niche. Hallett *et al.* (*13*) mitigated these potential biases by randomly subsetting the dataset to produce a uniform temporal distribution of site occurrences, leveling the number of presences between older and more recent time periods, whilst thinning the data based on geographic proximity. This approach was evaluated in (*27*), where, to keep the highest number of presences, only a spatial thinning was applied. The resulting reconstructions were compared to (*13*), obtaining qualitatively equivalent model reconstructions: testing the niche overlap between the two models using Schoener's D, it was found an overlap of 0.804 (p=0.01). Thus, we use the models from (*27*), as they include more data and extend the reconstructions to the last 300 k years. Briefly, in (*27*), the presences were subsetted by applying spatial thinning to avoid spatial autocorrelation (one presence for every 200 km radius was kept). Chronological uncertainty was accounted for by resampling each date 100 times from a truncated normal distribution defined by the mean and ±2σ. Each occurrence was then randomly paired with 200 randomly sampled locations matched by time (i.e. background in the SDMs) to capture the range of available climatic conditions. This procedure generated 100 independent datasets (i.e., "repeats"), each consisting of randomly selected and dated occurrences together with distinct sets of background points, allowing repeated analyses to account for this stochastic sampling. Then, a "time-varying-niche" General Additive Model (see below for details) was fitted using the R package *mgcv* (*97*). In the model, the interactions between environmental variable and time (fitted as tensor products) were considered, and the GAM formula was included as:

$$\begin{aligned} gam(obs \sim ti(bio07, k{=}4) &+ ti(bio08, k{=}4) + ti(bio10, k{=}4) \\ &+ ti(bio16, \mathrm{k}{=}4) + ti(lai, k{=}4) + ti(time\_bp, k{=}4) \\ &+ ti(time\_bp, bio07) + ti(time\_bp, bio08) + ti(time\_bp, bio10) \\ &+ ti(time\_bp, bio16) + ti(time\_bp, lai), \end{aligned}$$

data=*PA*, *family*='binomial')

To avoid overfitting, in all GAMs a maximum threshold set to 4 degrees of freedom of the splines , and residuals were tested for correct distribution, dispersion, and outliers (as in (*13*)) using Kolmogorov–Smirnov tests with the *DHARMa* R package (*98*).

<u>Including the human niche</u>
The niche area was divided into core areas (the smallest area including 90% of archaeological sites/presences) and extended areas (covering 95% and 99% of sites respectively). We chose an intermediate level of 0.95 to include the extension of humans across the landscape.

Firstly, we checked the spatial overlay of the human core niche against the maps of malaria stability index extent across time (see figure S5). Then, we compared the median of the index in the areas identified as core areas for humans against the median of the index in the areas outside the human range (i.e., areas identified as not suitable for human groups), considering upper and lower quantiles of 0.25 and 0.75. Desert areas (unsuitable for both humans and malaria vectors) were still included in this analysis.

<u>Species Distribution Models on other individual vector species</u>
Considering species complexes and groups (e.g., the *An. gambiae* complex and *An. funestus* group) is taxonomically appropriate, as this approach allowed us to integrate historical data with the greatest confidence and to focus on the dominant malaria vectors across Africa. However, in the past, other vectors might have played a role in the dynamics of malaria. We explored this possibility by performing SDMs on individual species, splitting the groups into 6 individual species and adding 3 species more zoophilic or opportunistic behaviour: *An. arabiensis*, *An. nili*, *An. gambiae* sensu strictu (ss), *An. funestus* ss, *An. melas*, *An. merus*, *An. coluzzii*, *An. moucheti* and *An. pharoensis*. Among these, *An. moucheti* is considered to affect both humans and primates (*99*);(*100*), *An. arabiensis* can opportunistically feed on cattle, and *An. nili*, despite being primarily anthropophilic, may occasionally feed on cattle too (*101*). Whilst the use of individual species increases the risk of misclassification, especially in older records, it allows us to test the robustness of our results by considering a broader range of vectors.

Importantly, disaggregating the SDMs to the species level had no qualitative impact on the results. Figure S6 shows the species habitat range for three time steps and fig. S7 the calculated malaria stability index for nine time steps (from 74 to 5 ka).

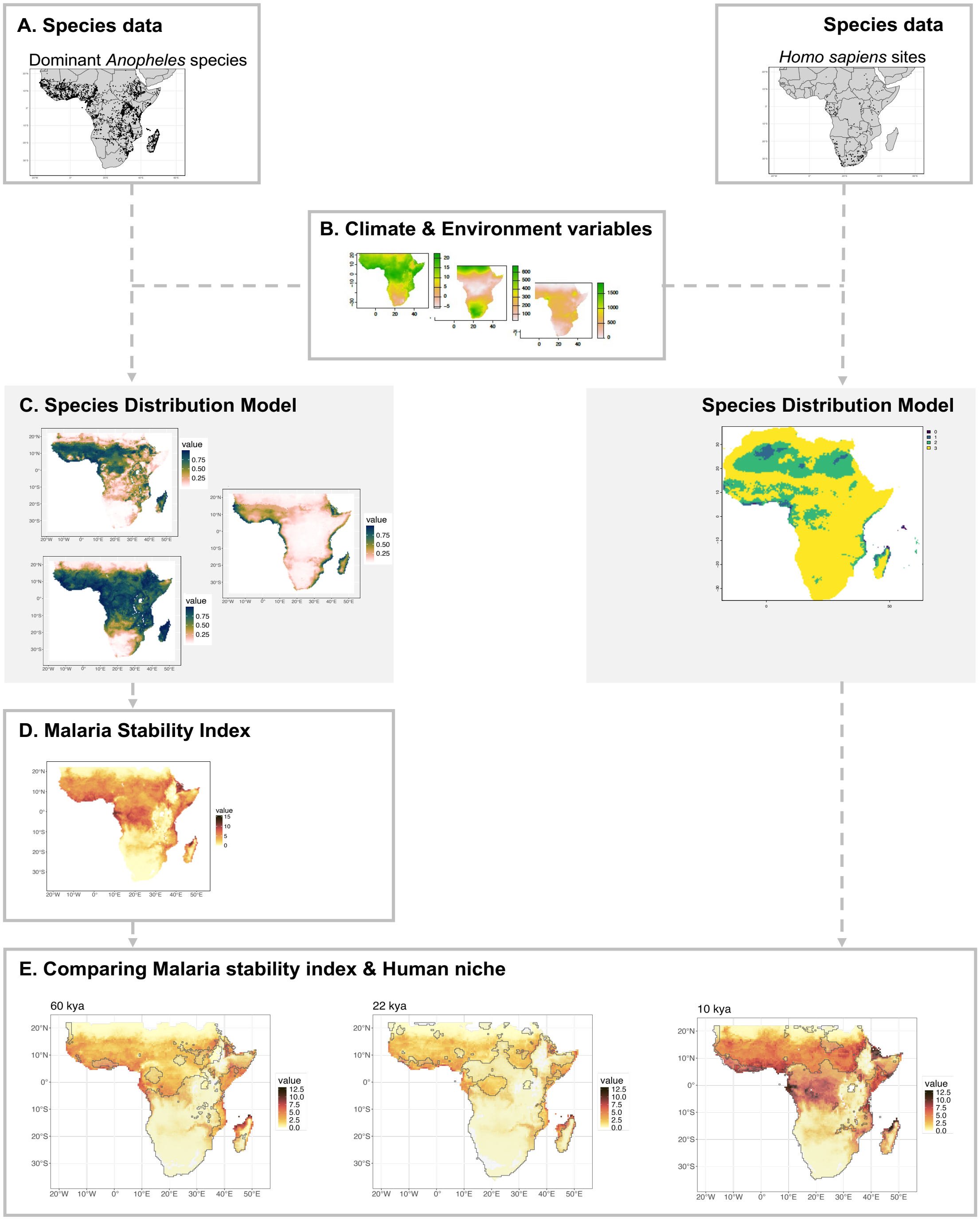

Fig. S1.
Overview of the methods and steps to model malaria through time. (A) starting with the species data points (observations in the present for each mosquito species or sites of *Homo sapiens* through time), we then included climatic and environmental reconstructions (B) to create independent reconstructions of the species ranges (Species Distribution Models) (C). The mosquito SDMs are then summarised and, including epidemiological information, (D) the malaria stability index is calculated. Finally, (E) we checked the spatial overlay of the human niche against the maps of malaria stability index extent across time.

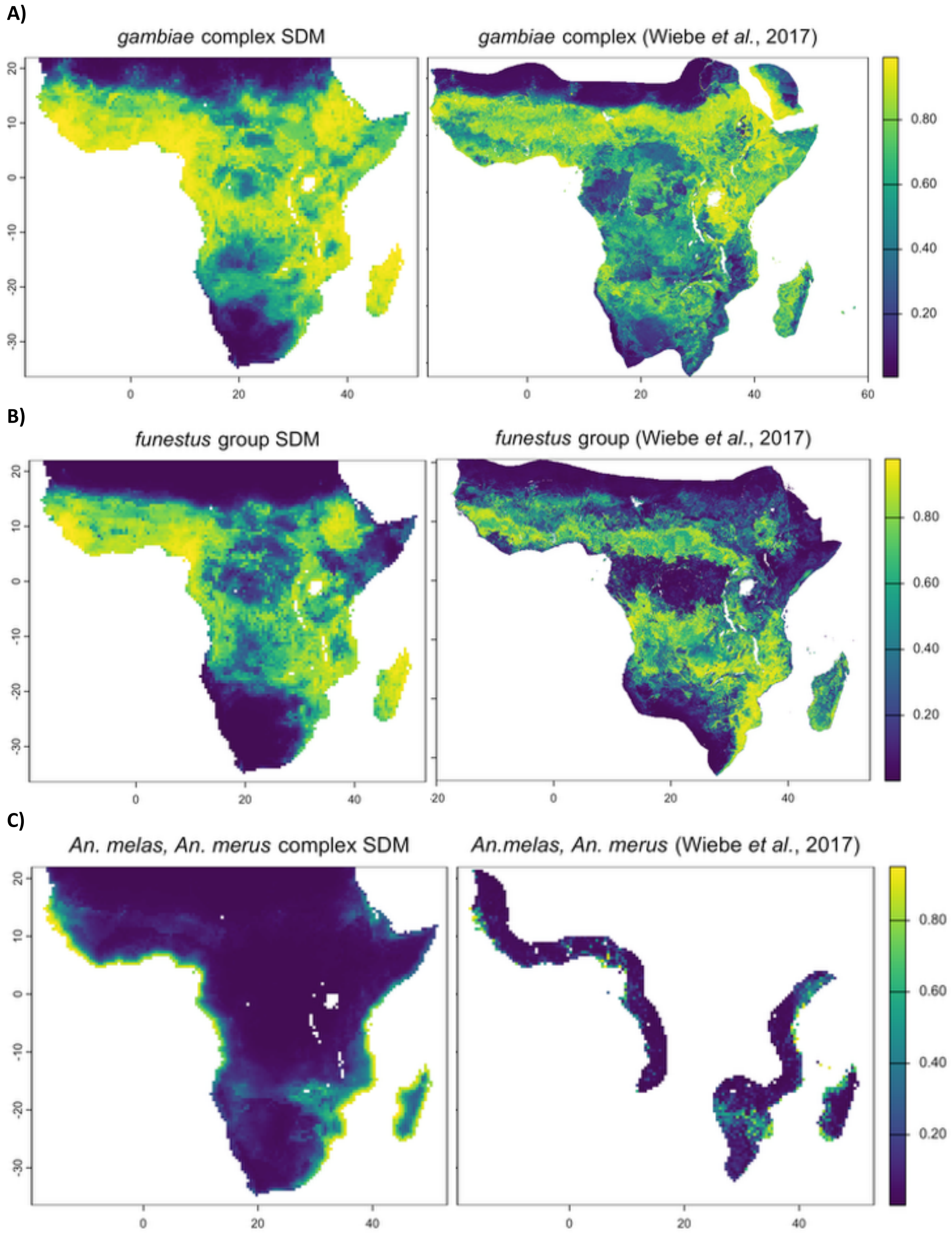

Fig. S2.
Accompanying SDM plots supporting niche overlap comparison between the current study (left) and Wiebe *et al.* (*2*) results (median) on the right. A) SDM of present suitability of *An. gambiae* complex; B) SDM of present suitability of *An. funestus* group; C) SDM of present suitability of *An. melas* and *An. merus*.

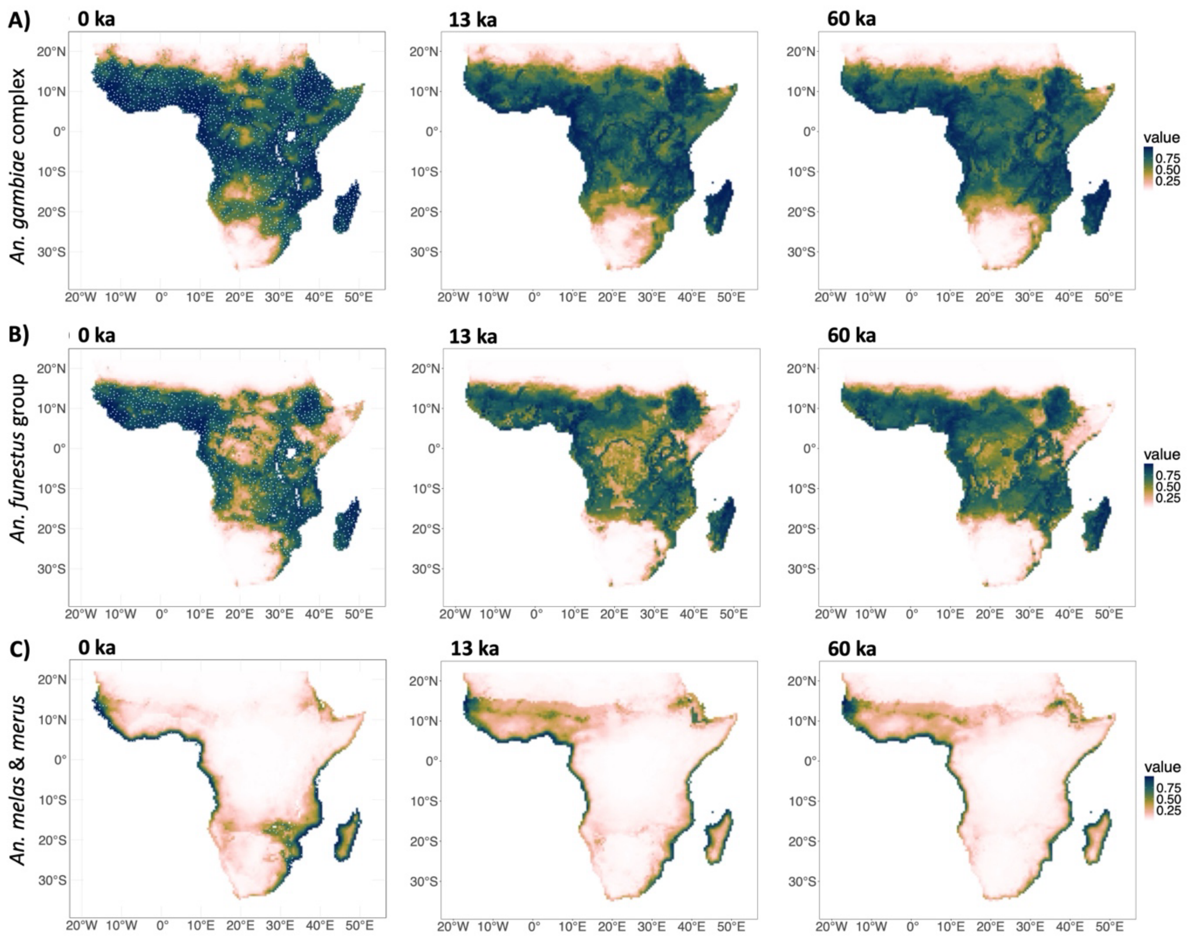

Fig. S3.

Anopheles vectors SDMs for the present and the past. These plots show the reconstructed niche of the studied vectors: A) *An. gambiae* complex at the present time (with observations included), at 13 and at 60 ka; B) *An. funestus* group at the present time (with observations included), at 13 and at 60 ka; and C) *An. melas* and *An. merus* at the present time (with observations included), at 13 and at 60 ka.

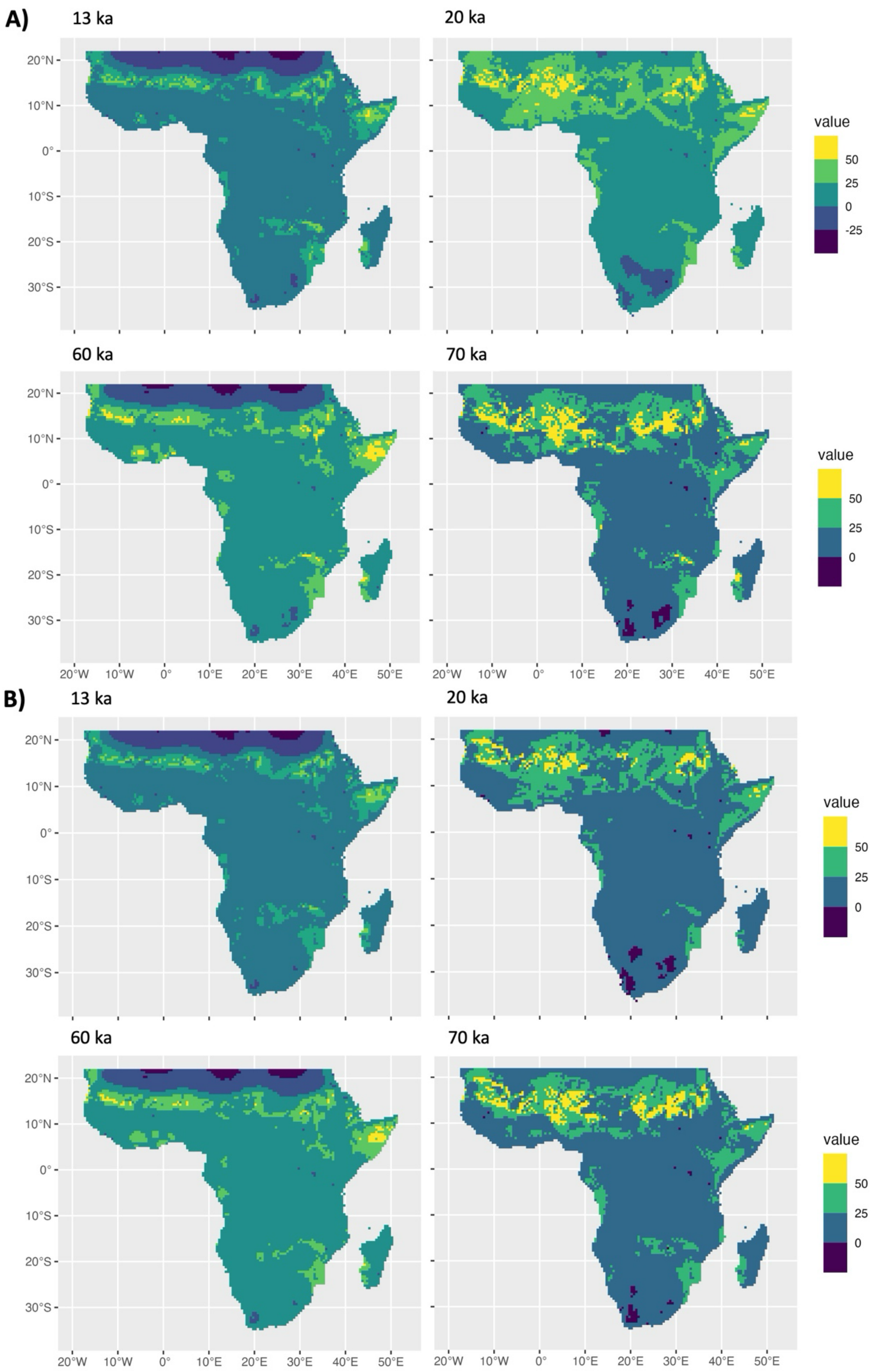
A)
13 ka
20 ka
60 ka
70 ka
B)
13 ka
20 ka
60 ka
70 ka
value
50
25
0
-25
20°N
10°N
0°
10°S
20°S
30°S
20°W
10°W
0°
10°E
20°E
30°E
40°E
50°E

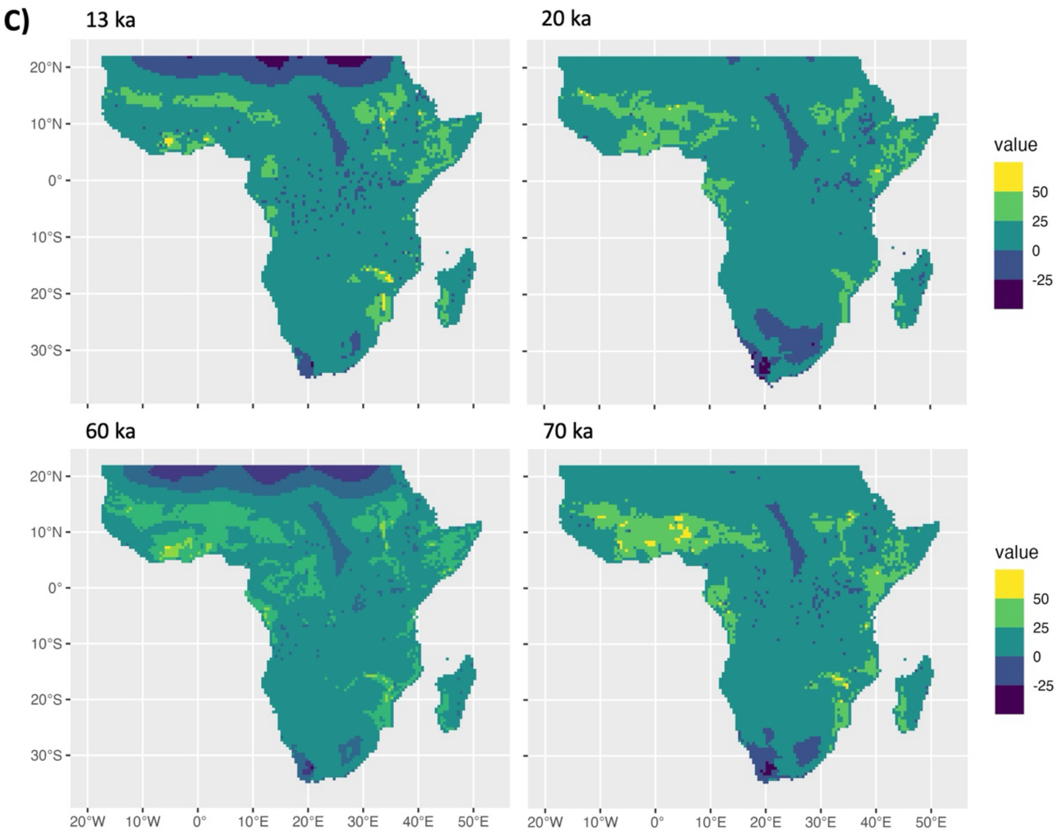

Fig. S4.
Multivariate environmental similarity surfaces (MESS) comparing present model conditions to past climate (positive values indicate conditions within the ranges used for training; negative values highlight areas with climatic conditions that are outside the training set, and thus might lead to incorrect extrapolation). A) *An. gambiae* complex; B) *An. funestus* group; C) *An. melas* and *An. merus.*

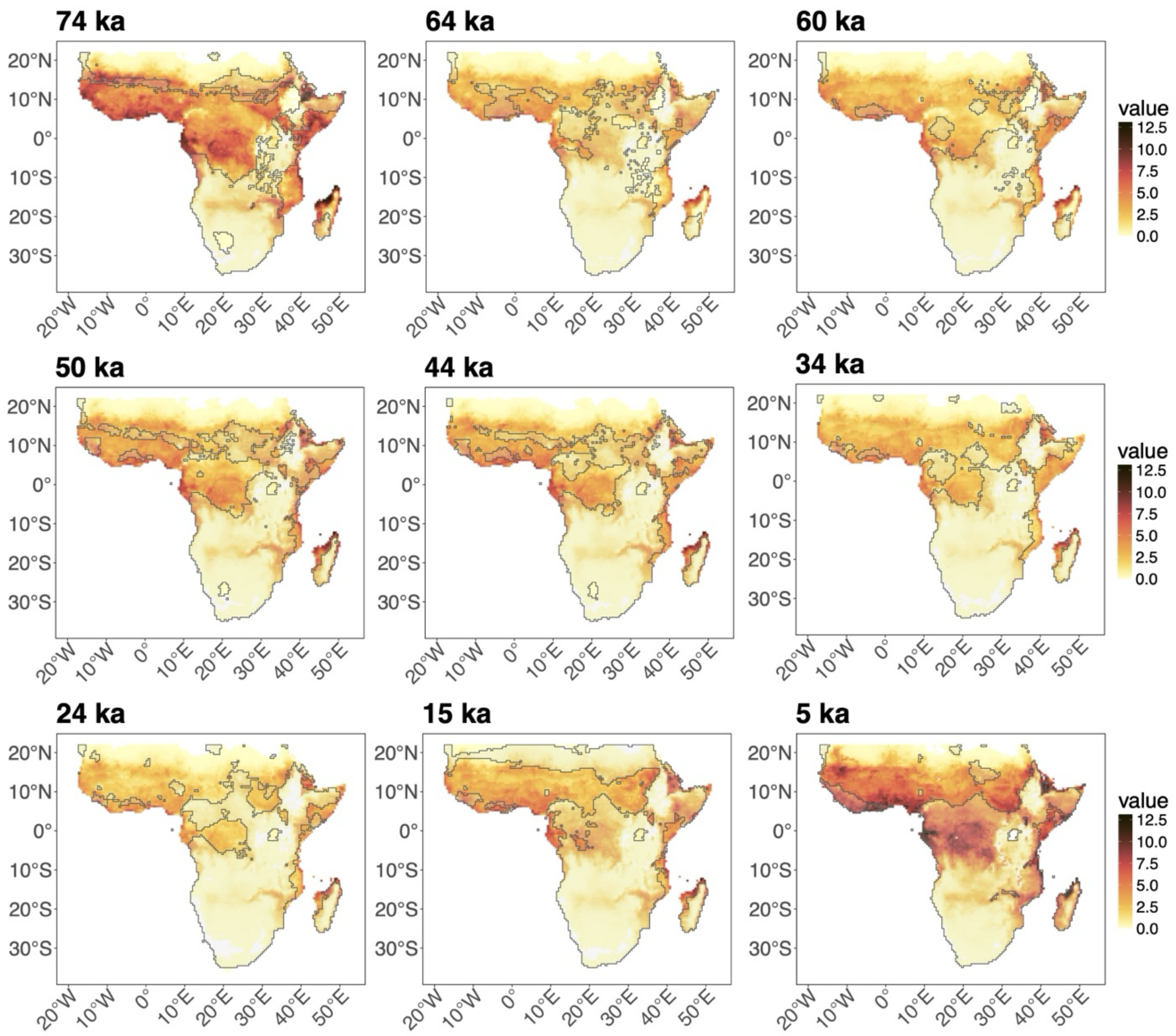

Fig. S5.
Comparing the extent of human niche and malaria stability index through time. These maps show the extent of the human niche (outlined in black) against the map of malaria stability index at nine time steps as an example: at 74 ka, 64 ka, 60 ka, 50 ka, 44 ka, 34 ka, 24 ka, 15 ka and 5 ka.

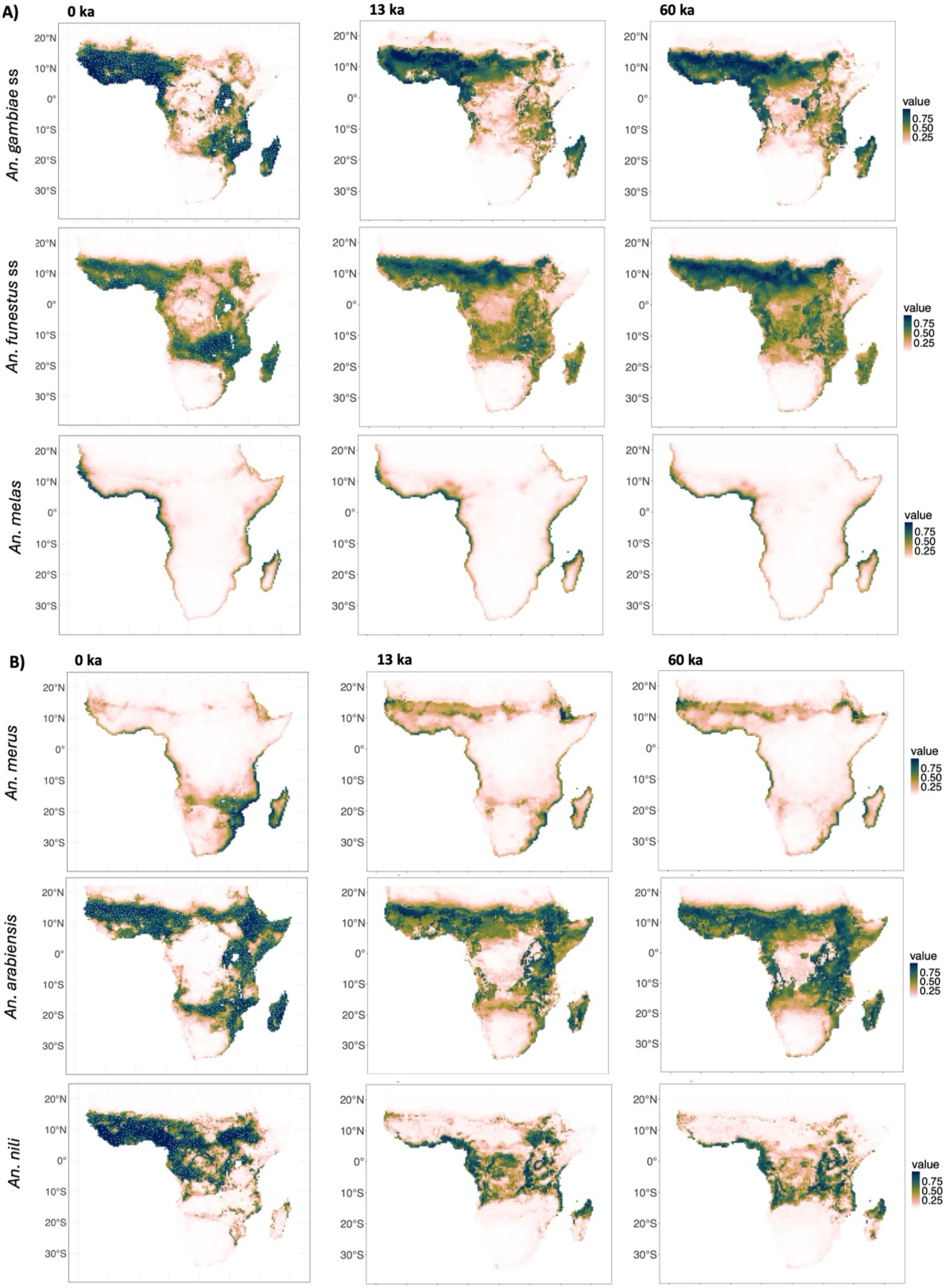

A)
0 ka
13 ka
60 ka
An. gambiae ss
An. funestus ss
An. melas
B)
An. merus
An. arabiensis
An. nili
20°N
10°N
0°
10°S
20°S
30°S
value
0.75
0.50
0.25

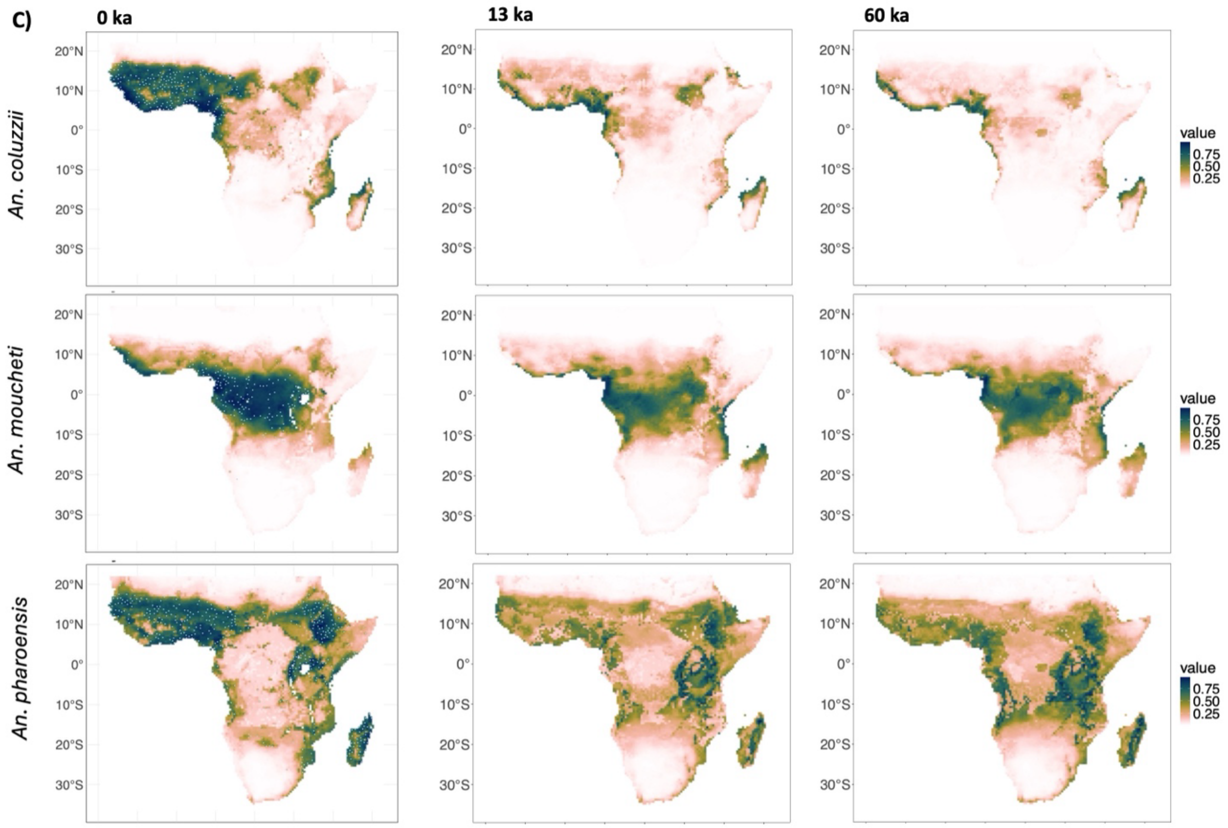

Fig. S6.
Species distribution models for 8 individual species at 0 ka (with observations included), 13 ka and 60 ka. *Anopheles* vectors SDMs for the present and the past. These plots show the reconstructed niche of the studied vectors: A) *An. gambiae* ss., *An. funestus* ss, *An. melas*; B) *An. merus, An. arabiensis, An. nili*, C) *An. coluzzii, An. moucheti, An. pharoensis*.

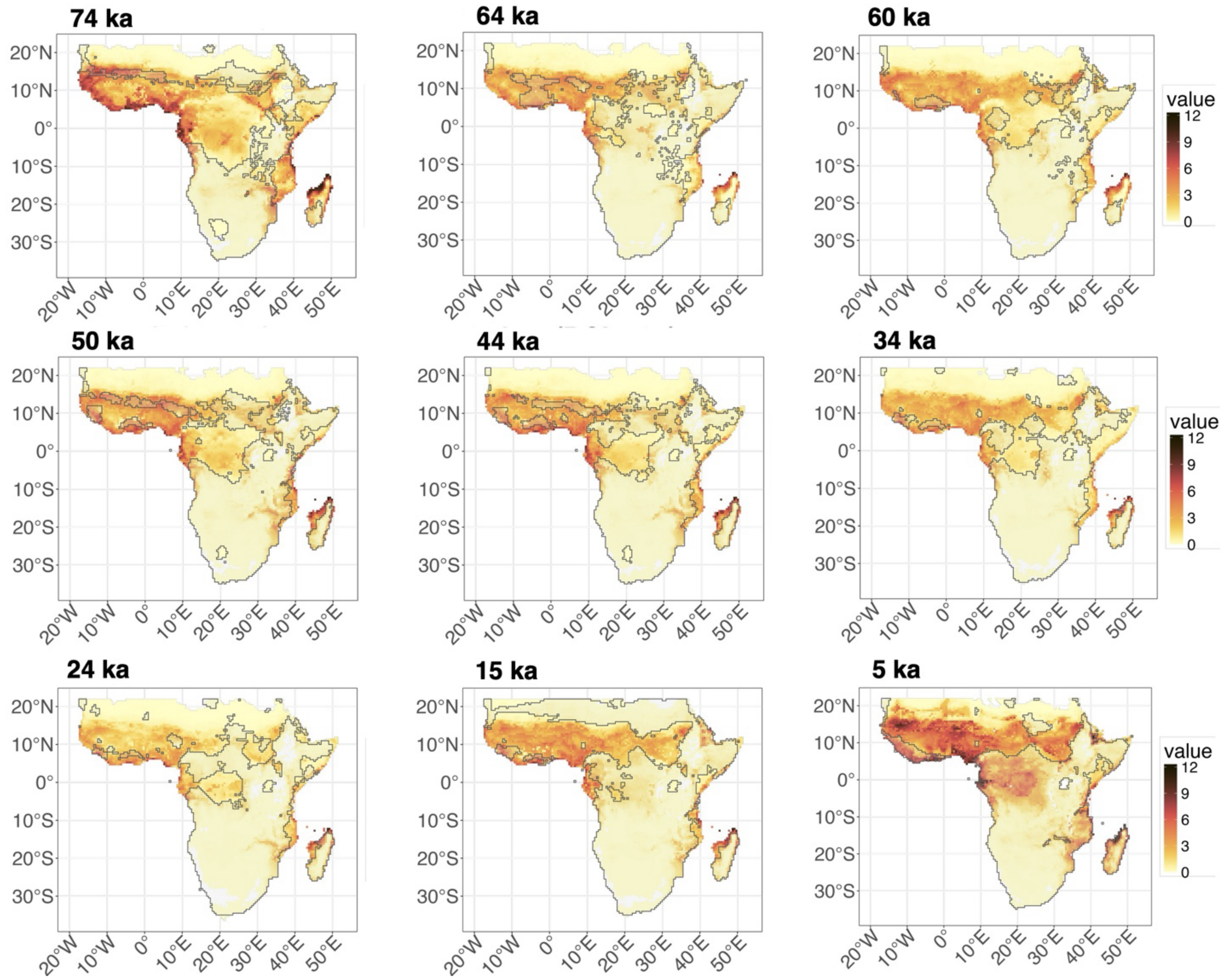

Fig. S7.
Comparing the extent of human niche and malaria stability index through time based on 9 individual species. These maps show the extent of the human niche (outlined in black) against the map of malaria stability index at nine time steps as an example: at 74 ka, 64 ka, 60 ka, 50 ka, 44 ka, 34 ka, 24 ka, 15 ka and 5 ka.

**Vectors in Wiebe *et al.* 2017 (*2*)**	**Kyalo *et al.* 2017 (*15*)**
An. gambiae complex: *An. gambiae* s.s., *An. arabiensis, An. coluzzi, An. amharicus, An. bwambae, An. quadriannulatus*	*gambiae* complex: *An. gambiae* s.s., SS M Form (*An colluzzi* or Mopti forms), SS S Form (savanah or Bamako forms), *An. arabiensis, An. bwambae*
An. melas & *An. merus*	*An. melas* & *An. merus*
An. funestus group*: *An. funestus* s.s., *An. aruni, An. brucei, An. confuses, An. fuscivenosus, An. leesoni, An. parensis, An. rivulorum, An. vaneedeni*	*An. funestus* s.l.: *An. funestus* s.s., *An. rivulorum, An. leesoni, An. parensis, An. vaneedeni*

Table S1.

Anopheles species included in this study and their sources. The reported presences are limited to sub-Saharan Africa and based on: Wiebe *et al.* (*2*); Kyalo *et al.* (*15*); * Expert information: additional observations (datapoints) for *An. funestus* s.s. based on Irish *et al.* (4) and Kyalo *et al.* (15). All data points are available for each species in tables S4-S6.

Analysis type	Vector in the study	Total	After thinning
Complexes	*An. gambiae* complex	14454	1219
	An. melas & *An. merus*	1080	87
	An. funestus group	5917	841
Individual species	*An. gambiae* ss	5691	491
	An. melas	716	43
	An. merus	364	44
	An. funestus ss	1453	204
	An. arabiensis	5741	578
	An. nili	822	306
	An. coluzzii	2417	221
	An. moucheti	499	150
	An. pharoensis	1889	430

Table S2.

Anopheles species included in this study before and after thinning.

Table S3. (separate file)

Table of variables selected and included for each *Anopheles* species. See file TableS1-S3_Anopheles_species.xlsx

Table S4. (separate file)

List of *An. merus* and *An. melas* observations used in this project. See file TableS4_An.melas.merus_observations.csv

Table S5. (separate file)

List of *An. funestus* group observations used in this project. See file TableS5_An.funestus.group_observations.csv

Table S6. (separate file)

List of *An. gambiae* complex observations used in this project. See file TableS6_An.gambiae.cplx_observations.csv

REFERENCES

1. A. Bergström, C. Stringer, M. Hajdinjak, E. M. L. Scerri, P. Skoglund, Origins of modern human ancestry. *Nature* **590**, 229–237 (2021).

2. A. P. Ragsdale, T. D. Weaver, E. G. Atkinson, E. G. Hoal, M. Möller, B. M. Henn, S. Gravel, A weakly structured stem for human origins in Africa. *Nature* **617**, 755–763 (2023).

3. E. M. L. Scerri, M. G. Thomas, A. Manica, P. Gunz, J. T. Stock, C. Stringer, M. Grove, H. S. Groucutt, A. Timmermann, G. P. Rightmire, F. d'Errico, C. A. Tryon, N. A. Drake, A. S. Brooks, R. W. Dennell, R. Durbin, B. M. Henn, J. Lee-Thorp, P. deMenocal, M. D. Petraglia, J. C. Thompson, A. Scally, L. Chikhi, Did our species evolve in subdivided populations across Africa, and why does it matter? *Trends Ecol. Evol.* **33**, 582–594 (2018).

4. P. Roberts, B. A. Stewart, Defining the 'generalist specialist' niche for Pleistocene *Homo sapiens*. *Nat. Hum. Behav.* **2**, 542–550 (2018).

5. E. Ben Arous, J. A. Blinkhorn, E. Elliott, C. Kiahtipes, C. D. N'zi, M. D. Bateman, M. Duval, P. Roberts, R. Patalano, A. F. Blackwood, K. Niang, E. A. Kouamé, E. Lebato, E. Hallett, J. N. Cerasoni, E. Scott, J. Ilgner, M. J. Alonso Escarza, F. Y. Guédé, E. M. L. Scerri, Humans in Africa's wet tropical forests 150 thousand years ago. *Nature* **640**, 402–407 (2025).

6. A. Timmermann, T. Friedrich, Late Pleistocene climate drivers of early human migration. *Nature* **538**, 92–95 (2016).

7. E. Y. Hallett, M. Leonardi, J. N. Cerasoni, M. Will, R. Beyer, M. Krapp, A. W. Kandel, A. Manica, E. M. L. Scerri, Major expansion in the human niche preceded out of Africa dispersal. *Nature* **644**, 115–121 (2025).

8. C. J. Houldcroft, S. Underdown, Infectious disease in the Pleistocene: Old friends or old foes? *Am. J. Biol. Anthropol.* **182**, 513–531 (2023).

9. G. Laval, S. Peyrégne, N. Zidane, C. Harmant, F. Renaud, E. Patin, F. Prugnolle, L. Quintana-Murci, Recent adaptive acquisition by African rainforest hunter-gatherers of the late Pleistocene sickle-cell mutation suggests past differences in malaria exposure. *Am. J. Hum. Genet.* **104**, 553–561 (2019).

10. M. Lipson, I. Ribot, S. Mallick, N. Rohland, I. Olalde, N. Adamski, N. Broomandkhoshbacht, A. M. Lawson, S. López, J. Oppenheimer, K. Stewardson, R. N. Asombang, H. Bocherens, N. Bradman, B. J. Culleton, E. Cornelissen, I. Crevecoeur, P. de Maret, F. L. M. Fomine, P. Lavachery, C. M. Mindzie, R. Orban, E. Sawchuk, P. Semal, M. G. Thomas, W. Van Neer, K. R. Veeramah, D. J. Kennett, N. Patterson, G. Hellenthal, C. Lalueza-Fox, S. MacEachern, M. E. Prendergast, D. Reich, Ancient West African foragers in the context of African population history. *Nature* **577**, 665–670 (2020).

11. G. D. Poznik, Y. Xue, F. L. Mendez, T. F. Willems, A. Massaia, M. A. Wilson Sayres, Q. Ayub, S. A. McCarthy, A. Narechania, S. Kashin, Y. Chen, R. Banerjee, J. L. Rodriguez-Flores, M. Cerezo, H. Shao, M. Gymrek, A. Malhotra, S. Louzada, R. Desalle, G. R. Ritchie, E. Cerveira, T. W. Fitzgerald, E. Garrison, A. Marcketta, D. Mittelman, M. Romanovitch, C. Zhang, X. Zheng-Bradley, G. R. Abecasis, S. A. McCarroll, P. Flicek, P. A. Underhill, L. Coin, D. R. Zerbino, F. Yang, C. Lee, L. Clarke, A. Auton, Y. Erlich, R. E. Handsaker, C. D. Bustamante, C. Tyler-Smith, Punctuated bursts in human male demography inferred from 1,244 worldwide Y-chromosome sequences. *Nat. Genet.* **48**, 593–599 (2016).

12. P. Skoglund, J. C. Thompson, M. E. Prendergast, A. Mittnik, K. Sirak, M. Hajdinjak, T. Salie, N. Rohland, S. Mallick, A. Peltzer, A. Heinze, I. Olalde, M. Ferry, E. Harney, M. Michel, K. Stewardson, J. I. Cerezo-Román, C. Chiumia, A. Crowther, E. Gomani-Chindebvu, A. O. Gidna, K. M. Grillo, I. T. Helenius, G. Hellenthal, R. Helm, M. Horton, S. López, A. Z. P. Mabulla, J. Parkington, C. Shipton, M. G. Thomas, R. Tibesasa, M. Welling, V. M. Hayes, D. J. Kennett, R. Ramesar, M. Meyer, S. Pääbo, N. Patterson, A. G. Morris, N. Boivin, R. Pinhasi, J. Krause, D. Reich, Reconstructing prehistoric African population structure. *Cell* **171**, 59–71.e21 (2017).

13. C. Ehret, Bantu expansions: Re-envisioning a central problem of early African history. *Int. J. Afr. Hist. Stud.* **34**, 5–41 (2001).

14. C. A. Fortes-Lima, C. Burgarella, R. Hammarén, A. Eriksson, M. Vicente, C. Jolly, A. Semo, H. Gunnink, S. Pacchiarotti, L. Mundeke, I. Matonda, J. K. Muluwa, P. Coutros, T. S. Nyambe, J. C. Cikomola, V. Coetzee, M. de Castro, P. Ebbesen, J. Delanghe, M. Stoneking, L. Barham, M. Lombard, A. Meyer, M. Steyn, H. Malmström, J. Rocha, H. Soodyall, B. Pakendorf, K. Bostoen, C. M. Schlebusch, The genetic legacy of the expansion of Bantu-speaking peoples in Africa. *Nature* **625**, 540–547 (2024).

15. R. Grollemund, S. Branford, K. Bostoen, A. Meade, C. Venditti, M. Pagel, Bantu expansion shows that habitat alters the route and pace of human dispersals. *Proc. Natl. Acad. Sci. U.S.A.* **112**, 13296–13301 (2015).

16. D. Shriner, C. N. Rotimi, Whole-genome-sequence-based haplotypes reveal single origin of the sickle allele during the Holocene Wet Phase. *Am. J. Hum. Genet.* **102**, 547–556 (2018).

17. WHO, "World Malaria Report 2024" (WHO, 2024).

18. M. Michel, E. Skourtanioti, F. Pierini, E. K. Guevara, A. Mötsch, A. Kocher, R. Barquera, R. A. Bianco, S. Carlhoff, L. Coppola Bove, S. Freilich, K. Giffin, T. Hermes, A. Hiß, F. Knolle, E. A. Nelson, G. U. Neumann, L. Papac, S. Penske, A. B. Rohrlach, N. Salem, L. Semerau, V. Villalba-Mouco, I. Abadie, M. Aldenderfer, J. F. Beckett, M. Brown, F. G. R. Campus, T. Chenghwa, M. Cruz Berrocal, L. Damašek, K. S. Duffett Carlson, R. Durand, M. Ernée, C. Fântăneanu, H. Frenzel, G. García Atiénzar, S. Guillén, E. Hsieh, M. Karwowski, D. Kelvin, N. Kelvin, A. Khokhlov, R. L. Kinaston, A. Korolev, K.-L. Krettek, M. Küßner, L. Lai, C. Look, K. Majander, K. Mandl, V. Mazzarello, M. McCormick, P. de Miguel Ibáñez, R. Murphy, R. E. Németh, K. Nordqvist, F. Novotny, M. Obenaus, L. Olmo-Enciso, P. Onkamo, J. Orschiedt, V. Patrushev, S. Peltola, A. Romero, S. Rubino, A. Sajantila, D. C. Salazar-García, E. Serrano, S. Shaydullaev, E. Sias, M. Šlaus, L. Stančo, T. Swanston, M. Teschler-Nicola, F. Valentin, K. Van de Vijver, T. L. Varney, A. Vigil-Escalera Guirado, C. K. Waters, E. Weiss-Krejci, E. Winter, T. C. Lamnidis, K. Prüfer, K. Nägele, M. Spyrou, S. Schiffels, P. W. Stockhammer, W. Haak, C. Posth, C. Warinner, K. I. Bos, A. Herbig, J. Krause, Ancient *Plasmodium* genomes shed light on the history of human malaria. *Nature* **631**, 125–133 (2024).

19. L. Wadley, C. Sievers, M. Bamford, P. Goldberg, F. Berna, C. Miller, Middle Stone Age bedding construction and settlement patterns at Sibudu, South Africa. *Science* **334**, 1388–1391 (2011).

20. T. J. Coulthard, J. A. Ramirez, N. Barton, M. Rogerson, T. Brücher, Were rivers flowing across the Sahara during the last interglacial? Implications for human migration through Africa. *PLOS ONE* **8**, e74834 (2013).

21. J. Elith, J. R. Leathwick, Species distribution models: Ecological explanation and prediction across space and time. *Annu. Rev. Ecol. Evol. Syst.* **40**, 677–697 (2009).

22. A. Kiszewski, A. Mellinger, A. Spielman, P. Malaney, S. E. Sachs, J. Sachs, A global index representing the stability of malaria transmission. *Am. J. Trop. Med. Hyg.* **70**, 486–498 (2004).

23. C. Padilla-Iglesias, Z. Xue, M. Leonardi, J. L. A. Paijmans, M. Colucci, A. Hovhannisyan, P. Maisano-Delser, J. Blanco-Portillo, A. G. Ioannidis, G. Lucarini, J. N. Cerasoni, A. W. Kandel, M. Will, E. Y. Hallett, K. Lupo, E. M. L. Scerri, I. Crevecoeur, L. Vinicius, A. B. Migliano, A. Manica, Pan-African metapopulation model explains *Homo sapiens* genetic and morphological evolution. bioRxiv 655514 [Preprint] (2025). https://doi.org/10.1101/2025.05.22.655514.

24. N. Ramankutty, J. A. Foley, Characterizing patterns of global land use: An analysis of global croplands data. *Global Biogeochem. Cycles* **12**, 667–685 (1998).

25. M. Tallavaara, E. K. Jørgensen, Why are population growth rate estimates of past and present hunter-gatherers so different? *Philos. Trans. R. Soc. Lond. B Biol. Sci.* **376**, 20190708 (2021).

26. M. B. Araújo, M. New, Ensemble forecasting of species distributions. *Trends Ecol. Evol.* **22**, 42–47 (2007).

27. A. Wiebe, J. Longbottom, K. Gleave, F. M. Shearer, M. E. Sinka, N. C. Massey, E. Cameron, S. Bhatt, P. W. Gething, J. Hemingway, D. L. Smith, M. Coleman, C. L. Moyes, Geographical distributions of African malaria vector sibling species and evidence for insecticide resistance. *Malar. J.* **16**, 85 (2017).

28. K. Tanabe, T. Mita, T. Jombart, A. Eriksson, S. Horibe, N. Palacpac, L. Ranford-Cartwright, H. Sawai, N. Sakihama, H. Ohmae, M. Nakamura, M. U. Ferreira, A. A. Escalante, F. Prugnolle, A. Björkman, A. Färnert, A. Kaneko, T. Horii, A. Manica, H. Kishino, F. Balloux, *Plasmodium falciparum* accompanied the human expansion out of Africa. *Curr. Biol.* **20**, 1283–1289 (2010).

29. M. Leonardi, F. Boschin, P. Boscato, A. Manica, Following the niche: the differential impact of the last glacial maximum on four European ungulates. *Commun. Biol.* **5**, 1038 (2022).

30. R. M. Beyer, M. Krapp, A. Manica, High-resolution terrestrial climate, bioclimate and vegetation for the last 120,000 years. *Sci. Data* **7**, 236 (2020).

31. M. Krapp, R. M. Beyer, S. L. Edmundson, P. J. Valdes, A. Manica, A statistics-based reconstruction of high-resolution global terrestrial climate for the last 800,000 years. *Sci. Data* **8**, 228 (2021).

32. T. Cousins, A. Scally, R. Durbin, A structured coalescent model reveals deep ancestral structure shared by all modern humans. *Nat. Genet.* **57**, 856–864 (2025).

33. M. Lipson, E. A. Sawchuk, J. C. Thompson, J. Oppenheimer, C. A. Tryon, K. L. Ranhorn, K. M. de Luna, K. A. Sirak, I. Olalde, S. H. Ambrose, J. W. Arthur, K. J. W. Arthur, G. Ayodo, A. Bertacchi, J. I. Cerezo-Román, B. J. Culleton, M. C. Curtis, J. Davis, A. O. Gidna, A. Hanson, P. Kaliba, M. Katongo, A. Kwekason, M. F. Laird, J. Lewis, A. Z. P. Mabulla, F. Mapemba, A. Morris, G. Mudenda, R. Mwafulirwa, D. Mwangomba, E. Ndiema, C. Ogola, F. Schilt, P. R. Willoughby, D. K. Wright, A. Zipkin, R. Pinhasi, D. J. Kennett, F. K. Manthi, N. Rohland, N. Patterson, D. Reich, M. E. Prendergast, Ancient DNA and deep population structure in sub-Saharan African foragers. *Nature* **603**, 290–296 (2022).

34. R. Barrett, C. W. Kuzawa, T. McDade, G. J. Armelagos, Emerging and re-emerging infectious diseases: The third epidemiologic transition. *Annu. Rev. Anthropol.* **27**, 247–271 (1998).

35. C. M. Lewis, M. Y. Akinyi, S. N. DeWitte, A. C. Stone, Ancient pathogens provide a window into health and well-being. *Proc. Natl. Acad. Sci. U.S.A.* **120**, e2209476119 (2023).

36. A. R. Omran, The epidemiologic transition. A theory of the epidemiology of population change. *Milbank Mem. Fund Q.* **49**, 509–538 (1971).

37. M. Sikora, E. Canteri, A. Fernandez-Guerra, N. Oskolkov, R. Ågren, L. Hansson, E. K. Irving-Pease, B. Mühlemann, S. Holtsmark Nielsen, G. Scorrano, M. E. Allentoft, F. Valeur Seersholm, H. Schroeder, C. Gaunitz, J. Stenderup, L. Vinner, T. C. Jones, B. Nystedt, K.-G. Sjögren, J. Parkhill, L. Fugger, F. Racimo, K. Kristiansen, A. K. N. Iversen, E. Willerslev, The spatiotemporal distribution of human pathogens in ancient Eurasia. *Nature* **643**, 1011–1019 (2025).

38. S. Taheri, M. A. González, M. J. Ruiz-López, S. Magallanes, S. Delacour-Estrella, J. Lucientes, R. Bueno-Marí, J. Martínez-de la Puente, D. Bravo-Barriga, E. Frontera, A. Polina, Y. Martinez-Barciela, J. M. Pereira, J. Garrido, C. Aranda, A. Marzal, I. Ruiz-Arrondo, J. A. Oteo, M. Ferraguti, R. Gutíerrez-López, R. Estrada, M. Miranda, C. Barceló, R. Morchón, T. Montalvo, L. Gangoso, F. Goiri, A. L. García-Pérez, S. Ruiz, B. Fernandez-Martinez, D. Gómez-Barroso, J. Figuerola, Modelling the spatial risk of malaria through probability distribution of *Anopheles maculipennis* s.l. and imported cases. *Emerg. Microbes Infect.* **13**, 2343911 (2024).

39. D. Kyalo, P. Amratia, C. W. Mundia, C. M. Mbogo, M. Coetzee, R. W. Snow, A geo-coded inventory of anophelines in the Afrotropical Region south of the Sahara: 1898-2016. *Wellcome Open Res.* **2**, 57 (2017).

40. M. P. Pierce, B. M. Worthington, S. Han, X.-B. Ni, Y. Liao, M. H. Shum, Y. Guan, E. C. Holmes, T. T. Lam, Phylogenomics redefines the evolutionary history of mosquitoes. *Proc. Natl. Acad. Sci. U.S.A.* **122**, e2519291122 (2025).

41. M. C. Fontaine, J. B. Pease, A. Steele, R. M. Waterhouse, D. E. Neafsey, I. V. Sharakhov, X. Jiang, A. B. Hall, F. Catteruccia, E. Kakani, S. N. Mitchell, Y.-C. Wu, H. A. Smith, R. R. Love, M. K. Lawniczak, M. A. Slotman, S. J. Emrich, M. W. Hahn, N. J. Besansky, Extensive introgression in a malaria vector species complex revealed by phylogenomics. *Science* **347**, 1258524 (2015).

42. Y. A. Afrane, M. Bonizzoni, G. Yan, in *Current Topics in Malaria*, J. R.-M. Alfonso, Ed. (IntechOpen, Rijeka, 2016), Chap. 20, pp. 473–490.

43. M. E. Sinka, M. J. Bangs, S. Manguin, M. Coetzee, C. M. Mbogo, J. Hemingway, A. P. Patil, W. H. Temperley, P. W. Gething, C. W. Kabaria, R. M. Okara, T. Van Boeckel, H. C. J. Godfray, R. E. Harbach, S. I. Hay, The dominant *Anopheles* vectors of human malaria in Africa, Europe and the Middle East: occurrence data, distribution maps and bionomic précis. *Parasit. Vectors* **3**, 117 (2010).

44. R. W. Snow, P. Amratia, C. W. Kabaria, A. M. Noor, K. Marsh, The changing limits and incidence of malaria in Africa: 1939-2009. *Adv. Parasitol.* **78**, 169–262 (2012).

45. M. Leonardi, E. Y. Hallett, R. B. Beyer, M. Krapp, A. Manica, pastclim 1.2: An R package to easily access and use paleoclimatic reconstructions. *Ecography* **2023**, e06481 (2023).

46. W. D. Gosling, M. Chevalier, M. L. Fischer, M. Holewijn, J. Finch, G. Gil-Romera, T. Hill, A. Houngnon, M. Leonardi, A. Manica, S. Kaboth-Bahr, A multi-model approach to the spatial and temporal characterization of the African Humid Period. *Quat. Int.* **744**, 109933 (2025).

47. K. K. Goldewijk, A. Beusen, J. Doelman, E. Stehfest, Anthropogenic land use estimates for the Holocene – HYDE 3.2. *Earth Syst. Sci. Data* **9**, 927–953 (2017).

48. G. Asner, J. Scurlock, J. Hicke, Global synthesis of leaf area index observations: Implications for ecological and remote sensing studies: Global leaf area index. *Glob. Ecol. Biogeogr.* **12**, 191–205 (2003).

49. S. R. Irish, D. Kyalo, R. W. Snow, M. Coetzee, Updated list of *Anopheles* species (Diptera: Culicidae) by country in the Afrotropical Region and associated islands. *Zootaxa* **4747**, zootaxa.4747.4743.4741 (2020).

50. M. Leonardi, M. Colucci, A. V. Pozzi, E. M. L. Scerri, A. Manica, tidysdm: Leveraging the flexibility of tidymodels for species distribution modelling in R. *Methods Ecol. Evol*. **15**, 1789–1795 (2024).

51. S. J. Phillips, M. Dudík, R. E. Schapire, Maxent software for modeling species niches and distributions (Version 3.4.1) (2024).

52. D. R. Roberts, V. Bahn, S. Ciuti, M. S. Boyce, J. Elith, G. Guillera-Arroita, S. Hauenstein,J. J. Lahoz-Monfort, B. Schröder, W. Thuiller, D. I. Warton, B. A. Wintle, F. Hartig, C. F. Dormann, Cross-validation strategies for data with temporal, spatial, hierarchical, orphylogenetic structure. *Ecography* **40**, 913-929 (2017).

53. R. Valavi, J. Elith, J. J. Lahoz-Monfort, G. Guillera-Arroita, blockCV: An r package for generating spatially or environmentally separated folds for k-fold cross-validation ofspecies distribution models. *Methods Ecol. Evol.* **10**, 225–232 (2019).

54. M. Krapp, R. Beyer, S. L. Edmundsson, P. J. Valdes, A. Manica, A comprehensive climate history of the last 800 thousand years. EarthArXiv (2019). https://doi.org/10.31223/osf.io/d5hfx.

55. J. J. Hublin, A. Ben-Ncer, S. E. Bailey, S. E. Freidline, S. Neubauer, M. M. Skinner, I. Bergmann, A. Le Cabec, S. Benazzi, K. Harvati, P. Gunz, New fossils from Jebel Irhoud, Morocco and the pan-African origin of *Homo sapiens*. *Nature* **546**, 289–292 (2017).

56. K. Harvati, C. Stringer, R. Grün, M. Aubert, P. Allsworth-Jones, C. A. Folorunso, The Later Stone Age calvaria from Iwo Eleru, Nigeria: Morphology and chronology. *PLOS ONE* **6**, e24024 (2011).

57. D. Richter, R. Grün, R. Joannes-Boyau, T. E. Steele, F. Amani, M. Rué, P. Fernandes, J. P. Raynal, D. Geraads, A. Ben-Ncer, J. J. Hublin, S. P. McPherron, The age of the hominin fossils from Jebel Irhoud, Morocco, and the origins of the Middle Stone Age. *Nature* **546**, 293–296 (2017).

58. E. M. L. Scerri, M. Will, The revolution that still isn't: The origins of behavioral complexity in *Homo sapiens*. *J. Hum. Evol.* **179**, 103358 (2023).

59. W. Rodríguez, O. Mazet, S. Grusea, A. Arredondo, J. M. Corujo, S. Boitard, L. Chikhi, The IICR and the non-stationary structured coalescent: Towards demographic inference with arbitrary changes in population structure. *Heredity* **121**, 663–678 (2018).

60. C. M. Schlebusch, H. Malmström, T. Günther, P. Sjödin, A. Coutinho, H. Edlund, A. R. Munters, M. Vicente, M. Steyn, H. Soodyall, M. Lombard, M. Jakobsson, Southern African ancient genomes estimate modern human divergence to 350,000 to 260,000 years ago. *Science* **358**, 652–655 (2017).

61. J. Blinkhorn, L. Timbrell, M. Grove, E. M. L. Scerri, Evaluating refugia in recent human evolution in Africa. *Philos. Trans. R. Soc. Lond. B Biol. Sci.* **377**, 20200485 (2022).

62. M. W. Blome, A. S. Cohen, C. A. Tryon, A. S. Brooks, J. Russell, The environmental context for the origins of modern human diversity: A synthesis of regional variability in African climate 150,000-30,000 years ago. *J. Hum. Evol.* **62**, 563–592 (2012).

63. S. Kaboth-Bahr, W. D. Gosling, R. Vogelsang, A. Bahr, E. M. L. Scerri, A. Asrat, A. S. Cohen, W. Düsing, V. Foerster, H. F. Lamb, M. A. Maslin, H. M. Roberts, F. Schäbitz, M. H. Trauth, Paleo-ENSO influence on African environments and early modern humans. *Proc. Natl. Acad. Sci. U.S.A.* **118**, e2018277118 (2021).

64. W. D. Gosling, E. M. L. Scerri, S. Kaboth-Bahr, The climate and vegetation backdrop to hominin evolution in Africa. *Philos. Trans. R. Soc. Lond. B Biol. Sci.* **377**, 20200483 (2022).

65. N. A. Drake, R. M. Blench, S. J. Armitage, C. S. Bristow, K. H. White, Ancient watercourses and biogeography of the Sahara explain the peopling of the desert. *Proc. Natl. Acad. Sci. U.S.A.* **108**, 458–462 (2011).

66. A. S. Cohen, J. R. Stone, K. R. M. Beuning, L. E. Park, P. N. Reinthal, D. Dettman, C. A. Scholz, T. C. Johnson, J. W. King, M. R. Talbot, E. T. Brown, S. J. Ivory, Ecological consequences of Early Late Pleistocene megadroughts in tropical Africa. *Proc. Natl. Acad. Sci. U.S.A.* **104**, 16422–16427 (2007).

67. E. D. Lorenzen, R. Heller, H. R. Siegismund, Comparative phylogeography of African savannah ungulates. *Mol. Ecol.* **21**, 3656–3670 (2012).

68. A. Kocher, L. Papac, R. Barquera, F. M. Key, M. A. Spyrou, R. Hübler, A. B. Rohrlach, F. Aron, R. Stahl, A. Wissgott, F. van Bömmel, M. Pfefferkorn, A. Mittnik, V. Villalba-Mouco, G. U. Neumann, M. Rivollat, M. S. van de Loosdrecht, K. Majander, R. I. Tukhbatova, L. Musralina, A. Ghalichi, S. Penske, S. Sabin, M. Michel, J. Gretzinger, E. A. Nelson, T. Ferraz, K. Nägele, C. Parker, M. Keller, E. K. Guevara, M. Feldman, S. Eisenmann, E. Skourtanioti, K. Giffin, G. A. Gnecchi-Ruscone, S. Friederich, V. Schimmenti, V. Khartanovich, M. K. Karapetian, M. S. Chaplygin, V. V. Kufterin, A. A. Khokhlov, A. A. Chizhevsky, D. A. Stashenkov, A. F. Kochkina, C. Tejedor-Rodríguez, Í. G.-M. de Lagrán, H. Arcusa-Magallón, R. Garrido-Pena, J. I. Royo-Guillén, J. Nováček, S. Rottier, S. Kacki, S. Saintot, E. Kaverzneva, A. B. Belinskiy, P. Velemínský, P. Limburský, M. Kostka, L. Loe, E. Popescu, R. Clarke, A. Lyons, R. Mortimer, A. Sajantila, Y. C. de Armas, S. T. H. Godoy, D. I. Hernández-Zaragoza, J. Pearson, D. Binder, P. Lefranc, A. R. Kantorovich, V. E. Maslov, L. Lai, M. Zoledziewska, J. F. Beckett, M. Langová, A. Danielisová, T. Ingman, G. G. Atiénzar, M. P. de Miguel Ibáñez, A. Romero, A. Sperduti, S. Beckett, S. J. Salter, E. D. Zilivinskaya, D. V. Vasil'ev, K. von Heyking, R. L. Burger, L. C. Salazar, L. Amkreutz, M. Navruzbekov, E. Rosenstock, C. Alonso-Fernández, V. Slavchev, A. A. Kalmykov, B. C. Atabiev, E. Batieva, M. A. Calmet, B. Llamas, M. Schultz, R. Krauß, J. Jiménez-Echevarría, M. Francken, S. Shnaider, P. de Knijff, E. Altena, K. Van de Vijver, L. Fehren-Schmitz, T. A. Tung, S. Lösch, M. Dobrovolskaya, N. Makarov, C. Read, M. Van Twest, C. Sagona, P. C. Ramsl, M. Akar, K. A. Yener, E. C. Ballestero, F. Cucca, V. Mazzarello, P. Utrilla, K. Rademaker, E. Fernández-Domínguez, D. Baird, P. Semal, L. Márquez-Morfín, M. Roksandic, H. Steiner, D. C. Salazar-García, N. Shishlina, Y. S. Erdal, F. Hallgren, Y. Boyadzhiev, K. Boyadzhiev, M. Küßner, D. Sayer, P. Onkamo, R. Skeates, M. Rojo-Guerra, A. Buzhilova, E. Khussainova, L. B. Djansugurova, A. Z. Beisenov, Z. Samashev, K. Massy, M. Mannino, V. Moiseyev, K. Mannermaa, O. Balanovsky, M.-F. Deguilloux, S. Reinhold, S. Hansen, E. P. Kitov, M. Dobeš, M. Ernée, H. Meller, K. W. Alt, K. Prüfer, C. Warinner, S. Schiffels, P. W. Stockhammer, K. Bos, C. Posth, A. Herbig, W. Haak, J. Krause, D. Kühnert, Ten millennia of hepatitis B virus evolution. *Science* **374**, 182–188 (2021).

69. B. Krause-Kyora, J. Susat, F. M. Key, D. Kühnert, E. Bosse, A. Immel, C. Rinne, S.-C. Kornell, D. Yepes, S. Franzenburg, H. O. Heyne, T. Meier, S. Lösch, H. Meller, S. Friederich, N. Nicklisch, K. W. Alt, S. Schreiber, A. Tholey, A. Herbig, A. Nebel, J. Krause, Neolithic and medieval virus genomes reveal complex evolution of hepatitis B. *eLife* **7**, e36666 (2018).

70. M. Fumagalli, F. Balloux, in *On Human Nature*, M. Tibayrenc, F. J. Ayala, Eds. (Academic Press, 2017), pp. 177–191.

71. A. L. Hughes, F. Verra, Very large long-term effective population size in the virulent human malaria parasite *Plasmodium falciparum*. *Proc. Biol. Sci.* **268**, 1855–1860 (2001).

72. F. B. Livingstone, Anthropological implications of sickle cell gene distribution in West Africa. *Am. Anthropol.* **60**, 533–562 (1958).

73. R. E. Howes, A. P. Patil, F. B. Piel, O. A. Nyangiri, C. W. Kabaria, P. W. Gething, P. A. Zimmerman, C. Barnadas, C. M. Beall, A. Gebremedhin, D. Ménard, T. N. Williams, D. J. Weatherall, S. I. Hay, The global distribution of the Duffy blood group. *Nat. Commun.* **2**, 266 (2011).

74. S. A. Tishkoff, R. Varkonyi, N. Cahinhinan, S. Abbes, G. Argyropoulos, G. Destro-Bisol, A. Drousiotou, B. Dangerfield, G. Lefranc, J. Loiselet, A. Piro, M. Stoneking, A. Tagarelli, G. Tagarelli, E. H. Touma, S. M. Williams, A. G. Clark, Haplotype diversity and linkage disequilibrium at human G6PD: Recent origin of alleles that confer malarial resistance. *Science* **293**, 455–462 (2001).

75. F. B. Piel, A. P. Patil, R. E. Howes, O. A. Nyangiri, P. W. Gething, T. N. Williams, D. J. Weatherall, S. I. Hay, Global distribution of the sickle cell gene and geographical confirmation of the malaria hypothesis. *Nat. Commun.* **1**, 104 (2010).

76. K. Esoh, A. Wonkam, Evolutionary history of sickle-cell mutation: Implications for global genetic medicine. *Hum. Mol. Genet.* **30**, R119–r128 (2021).

77. A. C. Allison, Protection afforded by sickle-cell trait against subtertian malareal infection. *Br. Med. J.* **1**, 290–294 (1954).

78. J. Lederberg, J. B. S. Haldane (1949) on infectious disease and evolution. *Genetics* **153**, 1–3 (1999).

79. S. L. Wiesenfeld, Sickle-cell trait in human biological and cultural evolution. Development of agriculture causing increased malaria is bound to gene-pool changes causing malaria reduction. *Science* **157**, 1134–1140 (1967).

80. J. G. Mears, H. M. Lachman, R. Cabannes, K. P. Amegnizin, D. Labie, R. L. Nagel, Sickle gene. Its origin and diffusion from West Africa. *J. Clin. Invest.* **68**, 606–610 (1981).

81. J. S. Wainscoat, The origin of mutant b-globin genes in human populations. *Acta Haematol.* **78**, 154–158 (2004).

82.1000G enomes Project Consortium, A. Auton, L. D. Brooks, R. M. Durbin, E. P. Garrison, H. M. Kang, J. O. Korbel, J. L. Marchini, S. M. Carthy, G. A. McVean, G. R. Abecasis, A global reference for human genetic variation. *Nature* **526**, 68–74 (2015).

83. International HapMap Consortium, The International HapMap Project. *Nature* **426**, 789–796 (2003).

84. R. A. Bentley, S. Carrignon, B. Gaydarska, J. Chapman, B. Buchanan, M. J. O'Brien, Modelling cultural responses to disease spread in Neolithic Trypillia mega-settlements. *J. R. Soc. Interface* **21**, 20240313 (2024).

85. T. S. Athni, M. S. Shocket, L. I. Couper, N. Nova, I. R. Caldwell, J. M. Caldwell, J. N. Childress, M. L. Childs, G. A. De Leo, D. G. Kirk, A. J. MacDonald, K. Olivarius, D. G. Pickel, S. O. Roberts, O. C. Winokur, H. S. Young, J. Cheng, E. A. Grant, P. M. Kurzner, S. Kyaw, B. J. Lin, R. C. Lopez, D. S. Massihpour, E. C. Olsen, M. Roache, A. Ruiz, E. A. Schultz, M. Shafat, R. L. Spencer, N. Bharti, E. A. Mordecai, The influence of vector-borne disease on human history: Socio-ecological mechanisms. *Ecol. Lett.* **24**, 829–846 (2021).

86. M. Ziegler, Malarial landscapes in Late Antique Rome and the Tiber Valley. *Landscapes* **17**, 139–155 (2016).

87. B. De Meillon, J. Gear, Malaria contracted on the Witwatersrand. *S. Afr. Med. J.* **13**, 309–312 (1939).

88. G. C. McCord, J. K. Anttila-Hughes, A malaria ecology index predicted spatial and temporal variation of malaria burden and efficacy of antimalarial interventions based on African serological data. *Am. Soc. Tropic. Med. Hygiene* **96**, 616–623 (2017).

89. P. Nie, C. He, J. Feng, Range dynamics of *Anopheles* mosquitoes in Africa suggest a significant increase in the malaria transmission risk. *Ecol. Evol.* **14**, e70059 (2024).

90. T. Lehmann, W. Hawley, H. Grebert, M. Danga, F. Atieli, F. Collins, The Rift Valley complex as a barrier to gene flow for *Anopheles gambiae* in Kenya. *J. Hered.* **90**, 613–621 (1999).

91. R. S. McCann, J.-P. Courneya, M. Donnelly, M. K. Laufer, T. Mzilahowa, K. Stewart, A. Miles, S. Takala-Harrison, T. D. O'Connor, Variation in spatial population structure in the *Anopheles gambiae* species complex. bioRxiv 595955 [Preprint] (2024). https://doi.org/10.1101/2024.05.26.595955.

92. R. J. Hijmans, S. E. Cameron, J. L. Parra, P. G. Jones, A. Jarvis, Very high resolution interpolated climate surfaces for global land areas. *Int. J. Climatol.* **25**, 1965–1978 (2005).

93. M. Barbet-Massin, F. Jiguet, C. H. Albert, W. Thuiller, Selecting pseudo-absences for species distribution models: How, where and how many? *Methods Ecol. Evol.* **3**, 327–338 (2012).

94. I. W. Renner, J. Elith, A. Baddeley, W. Fithian, T. Hastie, S. J. Phillips, G. Popovic, D. I. Warton, Point process models for presence-only analysis. *Methods Ecol. Evol.* **6**, 366–379 (2015).

95. C. Padilla-Iglesias, M. Grove, J. Blinkhorn, Ecological drivers of hunter-gatherer lithic technology from the Middle and Later Stone Age in Central Africa. *Quat. Sci. Rev.* **322**, 108390 (2023).

96. C. Padilla-Iglesias, L. M. Atmore, J. Olivero, K. Lupo, A. Manica, E. Arango Isaza, L. Vinicius, A. B. Migliano, Population interconnectivity over the past 120,000 years explains distribution and diversity of Central African hunter-gatherers. *Proc. Natl. Acad. Sci. U.S.A.* **119**, e2113936119 (2022).

97. E. R. Crema, A. Bevan, Inference from large sets of radiocarbon dates: Software and methods. *Radiocarbon* **63**, 23–39 (2021).

98. P. J. Reimer, W. E. N. Austin, E. Bard, A. Bayliss, P. G. Blackwell, C. Bronk Ramsey, M. Butzin, H. Cheng, R. L. Edwards, M. Friedrich, P. M. Grootes, T. P. Guilderson, I. Hajdas, T. J. Heaton, A. G. Hogg, K. A. Hughen, B. Kromer, S. W. Manning, R. Muscheler, J. G. Palmer, C. Pearson, J. van der Plicht, R. W. Reimer, D. A. Richards, E. M. Scott, J. R. Southon, C. S. M. Turney, L. Wacker, F. Adolphi, U. Büntgen, M. Capano, S. M. Fahrni, A. Fogtmann-Schulz, R. Friedrich, P. Köhler, S. Kudsk, F. Miyake, J. Olsen, F. Reinig, M. Sakamoto, A. Sookdeo, S. Talamo, The IntCal20 Northern Hemisphere radiocarbon age calibration curve (0–55 cal kBP). *Radiocarbon* **62**, 725–757 (2020).

99. S. N. Wood, N. H. Augustin, GAMs with integrated model selection using penalized regression splines and applications to environmental modelling. *Ecol. Model.* **157**, 157–177 (2002).

100. F. Hartig, DHARMa: Residual Diagnostics for Hierarchical (Multi-Level / Mixed) Regression Models (2022). https://CRAN.R-project.org/package=DHARMa.

101. C. Paupy, B. Makanga, B. Ollomo, N. Rahola, P. Durand, J. Magnus, E. Willaume, F. Renaud, D. Fontenille, F. Prugnolle, *Anopheles moucheti* and *Anopheles vinckei* are candidate vectors of ape *Plasmodium* parasites, including *Plasmodium praefalciparum* in Gabon. *PLOS ONE* **8**, e57294 (2013).

102. M. T. Gillies, B. De Meillon, *The Anophelinae of Africa South of the Sahara: Ethiopian Zoogeographical Region* (South African Institute for Medical Research, 1968).

103. C. Antonio-Nkondjio, H. Awono-Ambene, D. Fontenille, J. Meunier, La presence des bovins comme hotes alternatifs peut-elle modifier le comportement trophique des vecteurs du paludisme en zone de foret? *Bull. liaison doc.-OCEAC* **1**, 7–12 (2009). [Can the presence of cattle as alternative hosts alter the feeding behavior of malaria vectors in forested areas?].
